# WRKY Transcription Factor *OsWRKY29* Represses Seed Dormancy in Rice by Weakening Abscisic Acid Response

**DOI:** 10.3389/fpls.2020.00691

**Published:** 2020-05-27

**Authors:** Chunlei Zhou, Qibing Lin, Jie Lan, Tianyu Zhang, Xi Liu, Rong Miao, Changling Mou, Thanhliem Nguyen, Jiachang Wang, Xiao Zhang, Liang Zhou, Xingjie Zhu, Qian Wang, Xin Zhang, Xiuping Guo, Shijia Liu, Ling Jiang, Jianmin Wan

**Affiliations:** ^1^National Key Laboratory for Crop Genetics and Germplasm Enhancement, Nanjing Agricultural University, Nanjing, China; ^2^National Key Facility for Crop Gene Resources and Genetic Improvement, Institute of Crop Science, Chinese Academy of Agricultural Sciences, Beijing, China; ^3^Department of Biology and Agricultural Engineering, Quynhon University, Quynhon, Vietnam

**Keywords:** ABA signaling, *OsABF1*, *Oryza sativa*, seed dormancy, *OsVP1*

## Abstract

For efficient plant reproduction, seed dormancy delays seed germination until the environment is suitable for the next generation growth and development. The phytohormone abscisic acid (ABA) plays important role in the induction and maintenance of seed dormancy. Previous studies have identified that WRKY transcription factors can regulate ABA signaling pathway. Here, we identified an *Oswrky29* mutant with enhanced dormancy in a screen of T-DNA insertion population. OsWRKY29 is a member of WRKY transcription factor family which located in the nuclear. The genetic analyses showed that both knockout and RNAi lines of *OsWRKY29* had enhanced seed dormancy whereas its overexpression lines displayed reduced seed dormancy. When treated with ABA, *OsWRKY29* knockout and RNAi lines showed greater sensitivity than its overexpression lines. In addition, the expression levels of ABA positive response factors *OsVP1* and *OsABF1* were higher in the *OsWRKY29* mutants but were lower in its overexpression lines. Further assays showed that OsWRKY29 could bind to the promoters of *OsABF1* and *OsVP1* to inhibit their expression. In summary, we identified a new ABA signaling repressor OsWRKY29 that represses seed dormancy by directly downregulating the expression of *OsABF1* and *OsVP1*.

## Introduction

The core role of seeds is to reproduce offspring. Seed plants have evolved diverse strategies to ensure survival of their offspring. Among those strategies, seed dormancy prevents immediate germination until appropriate seasonal conditions favor their survival and proliferation (Koornneef et al., 2002; Shu et al., 2016; Penfield, 2017). Pre-harvest sprouting is one of the major problems in cereal production, damaging about 20% of the acreage of hybrid rice in southern China (Hu et al., 2003). By preventing pre-harvest sprouting, seed dormancy can maintain crop yield and quality (Liu et al., 2019). Therefore, it is important to understand and exploit the underlying molecular mechanisms.

The phytohormones abscisic acid (ABA) play major roles in regulating seed dormancy: ABA accumulates in the developing embryo, inducing and maintaining seed dormancy (Finkelstein et al., 2008; Nambara et al., 2010). ABA works through a complex signaling pathway, most components of which are identified. The core components include ABA receptors PYR/PYL/RCAR, negative regulators (protein phosphatase PP2Cs), positive effectors (protein kinase SnRK2s), and various downstream transcription factors such as *ABF1, ABF2*, *ABF3*, *ABF4*, and *ABI5* (Finkelstein and Lynch, 2000; Kang et al., 2002; Kim et al., 2004; Fujii et al., 2007; Ma et al., 2009; Park et al., 2009; Yoshida et al., 2010; Fuchs et al., 2014; Yu et al., 2015). In the absence of ABA, PP2Cs dephosphorylate SnRK2s to inhibit their kinase activity. In the presence of ABA, PYR/PYL/RCAR receptors bind to ABA and PP2C, thus unleashing the repression of PP2Cs on the activity of SnRK2s (Hauser et al., 2011). Activated SnRK2s subsequently phosphorylated downstream ABRE-binding protein/ABRE-binding (AREB/ABF) transcription factors to activate ABA responsive genes. Among AREB/ABF transcription factors, *ABA insensitive 5* (*ABI5*), a member of the basic leucine zipper transcription (bZIP) factor family, plays a central role in regulating ABA-responsive genes in seeds of *Arabidopsis* (Finkelstein and Lynch, 2000). In addition, ABI3, a B3-type APETALA2 domain transcription factor, have been reported to interact with ABI5 and acts upstream of ABI5 to induce the expression of ABA-responsive genes, thus repressing seed germination in Arabidopsis (Lopez-Molina et al., 2002). The function of *ABI3* in inducing seed dormancy was likely conserved in plants because *CnABI3* in Chamaecyparis nootkatensis (Zeng et al., 2003), *VIVIPAROUA 1* (*VP1*) in maize (Robichaud et al., 1979) and *OsVP1* in rice (Gao et al., 2010) all can induce seed dormancy. A bZIP transcription factor, *ABF1*, is also an important downstream gene in ABA signaling. In Arabidopsis, the *abf1* mutant seed exhibited less dormancy and hyposensitive to ABA (Sharma et al., 2011). In rice, the upregulated expression of some ABA-responsive genes was suppressed in *Osabf1* mutants (Hossain et al., 2010). Overexpression lines of *OsABF1* (*OsbZIP12*) were hypersensitive to ABA (Joo et al., 2014).

The WRKY family, one of the largest transcription factor families in plants, is involved in regulating many plant processes such as pathogen defense, stress responses and plant development (Wang et al., 2010; Chen et al., 2013; Hu et al., 2013; Li et al., 2016). In *Oryza sativa* spp. *japonica*, the WRKY transcription factor superfamily consists of an estimated 98 members that fall into four major structural groups based on number of WRKY domains and specific features of their zinc finger-like motifs (Ross et al., 2007). Most WRKY proteins have a high binding affinity to the DNA sequence (T)(T)TGAC(C/T), which is known as W-box (Ross et al., 2007). Although WRKY transcription factors can bind to the same W-box they still have functional differences in regulating seed dormancy by modulating the ABA signaling pathway. Some WRKY proteins promote seed dormancy by positively modulating ABA-responsive genes. For example, *AtWRKY2* promotes expression of *ABI3* and *ABI5*; meanwhile, ABA-induced *AtWRKY2* transcription requires the participation of *ABI3* and *ABI5* (Jiang and Yu, 2009), suggesting a positive feedback loop between *AtWRKY2* and *ABI3* or *ABI5* in regulating ABA response. *AtWRKY63* activates ABA-responsive genes such as *ABF2*, *RD29A*, and *COR47* (Ren et al., 2010). AtWRKY41 protein is reported to positively regulate *ABI3* expression (Ding et al., 2014). AtWRKY6 promotes ABA signaling by direct downregulating the expression of *RAV1*, which is a repressor of *ABI3*, *ABI4* and *ABI5* expression (Huang et al., 2016). OsWRKY72 and OsWRKY77 activate promoter activity of ABA-induced gene *HVA22* in barley aleurone cells (Xie et al., 2005). Some WRKY proteins repress seed dormancy by negatively modulating ABA-responsive genes. For example, AtWRKY18, AtWRKY40 and AtWRKY60 negatively regulate ABA signaling by directly repressing the expression of ABA-responsive genes, *MYB2*, *ABI4*, *ABF4*, *ABI5*, *DREB1A*, and *RAB18* (Shang et al., 2010). OsWRKY24 and OsWRKY45 repress ABA-induced *HVA22* expression in barley aleurone cells (Xie et al., 2005). However, the exact role of WRKY transcription factors in ABA response is obscure and their possible roles are often overlooked in studies of ABA signaling (Rushton et al., 2012). Thus, more WRKY genes need to be studied for better understood of their exact role in regulating ABA signaling.

In this study, we identified a new WRKY transcription factor, *OsWRKY29*, which negatively regulates seed dormancy in rice. Knockout and RNA interference of *OsWRKY29* enhanced seed dormancy whereas its overexpression reduced seed dormancy. The germinating seeds of *OsWRKY29* knockout and RNAi lines displayed hypersensitivity whereas these of *OsWRKY29*-overexpression lines displayed hyposensitivity to ABA. Compared with wild type, the expression of ABA positive response genes *OsABF1* and *OsVP1* was increased in *OsWRKY29* mutants but reduced in *OsWRKY29*-overexpression lines. Further analyses showed that OsWRKY29 directly binds to W-boxes in the promoters of *OsABF1* and *OsVP1* to repress their expression. These results suggest that *OsWRKY29* represses seed dormancy by weakening ABA response.

## Materials and Methods

### Plant Materials and Growth Conditions

*Oswrky29* mutant (2D-41043L) screened from a set of activation-tagged T-DNA insertion lines [in cultivar (cv.) Hwayoung (Hy)] (Jeong et al., 2002) was kindly provided by Prof. Gynheung An (Department of Plant Systems Biotech, Kyung Hee University, South Korea). All plants were grown at two field sites, Tuqiao in Nanjing and Sanya in Hainan province.

### Evaluation of Seed Dormancy

Dormancy levels were assessed according to the method of Wan et al. (2005), but the date of seeds harvest and the number of experimental samples were modified. Filled intact grains for evaluation of dormancy were collected at 45 days post heading. Freshly harvested seeds were placed on double sheets of filter paper in a 9 cm diameter Petri dish, moistened with distilled water, and maintained at 30°C and 100% relative humidity for 5 days. Each plant was tested in three replicates. 30 seeds were measured in each replicate. These seeds in three replicates are from the same harvest of the same plant. A germinated seed was declared when the plumule or radicle reached half the seed length. The percentage of germinated seeds on the 5th day was used as a measure of dormancy. For detection of sensitivity to ABA the harvested seeds were exposed to 50°C for 72 h to break dormancy (Lu et al., 2011). The heat-treated seeds (dormancy released stage) were used for ABA sensitivity tests. Seeds were germinated in 1, 3, and 5μM ABA solutions and the germination percentage was counted on the 5th or 7th day. Each plant was tested in three replicates. Thirty seeds were measured in each replicate.

### Primer Design, RNA Extraction and RT-qPCR Analysis

Primer Premier 5.0 software was used to design primers, and the primers used in confirming T-DNA insertion sites are listed in Supplementary Table S1. Total RNA was extracted from tissues using a RNAprep Pure Plant Kit (Tiangen Biotech Co. Ltd., Beijing, China) according to the manufacturer’s instructions. First-strand cDNA was synthesized using a PrimeScript^TM^ II 1st Strand cDNA Synthesis Kit (TaKaRa, Dalian). Synthesized cDNAs were used for RT-qPCR with a SYBR Premix Ex Taq kit (TaKaRa, Dalian, China) on an ABI 7500 real-time PCR system (Thermo Fisher Scientific, Waltham, MA, United States). Determination of relative changes in gene expression levels was based on three biological replicates. Primers were designed using the GenScript real-time PCR (TaqMan) primer design tool^1^ (Primers are listed in Supplementary Table S1). The rice UBQ5 gene (LOC_Os03g13170) was used to normalize cDNA quantity.

### Vector Construction and Plant Transformation

In order to knock out the *OsWRKY29* gene, we cloned 20 bp gene-specific spacer sequences into the sgRNA-Cas9 vector and then introduced it into calli of cv. Hwayoung (primer pair *OsWRKY29*-CRISPR in Supplementary Table S1) by Agrobacterium-mediated transformation. We identified positive transgenic individuals by sequencing.

*OsWRKY29* RNA interference plants were generated by amplification of 228 bp cDNA of *OsWRKY29* using primer pair *OsWRKY29*-RNAi-*Sac*I and *OsWRKY29*-RNAi-*Sna*BI (Supplementary Table S1). We fused *OsWRKY29*-RNAi-*Sac*I into the *Sac*I restriction site of the LH-FAD1390RNAi binary vector to construct an *OsWRKY29-RNAi-Sac*I vector, then fused *OsWRKY29-RNAi-Sna*BI into the *Sna*BI restriction site of the *OsWRKY29-RNAi-Sac*I vector to construct an *OsWRKY29-RNAi* vector, which was introduced into calli of Nipponbare by Agrobacterium-mediated transformation.

*OsWRKY29*-overexpressing plants were generated by amplification of full-length CDS of *OsWRKY29* using primer pair *OsWRKY29*-OX (Supplementary Table S1) and fused into the *Kpn*I/*Bam*HI site of the pCUBi1390 binary vector; and the resulting *UBi: OsWRKY29-overexpression* construct was inserted into calli of Nipponbare by Agrobacterium-mediated transformation.

### Subcellular Localization

Full-length CDS of *OsWRKY29* for subcellular localization of OsWRKY29 protein was inserted into the *Xba*I/*Bam*HI restriction site upstream of GFP in expression vector pAN580 driven by a double CaMV35S promoter to generate the *OsWRKY29-GFP* construct (primer pair *OsWRKY29*-GFP in Supplementary Table S1). The full-length CDS was also inserted into the *Bgl*II/*Pst*I restriction site downstream of GFP in expression vector pAN580 driven by a double CaMV35S promoter to generate a *GFP-OsWRKY29* construct (primer pair GFP-*OsWRKY29* in Supplementary Table S1). The *OsWRKY29-GFP* and *GFP-OsWRKY29* plasmids were introduced into rice protoplasts according to a published protocol (Zhang et al., 2011). Fluorescence of GFP was observed using a laser scanning confocal microscope (LSM 700; Zeiss).

### Transcription Activity Assay

Two reporters were used in Transcription activity assays. One contained the firefly luciferase (LUC) gene fused with 5 × GAL4 binding site, and the other was a renilla luciferase (REN) gene under control of CaMV35S promoter as the internal control. Full-length of OsWRKY29 was fused with the GAL4-BD and driven by a CaMV35S promoter to generate effector. The plasmids with effector (*GAL4-BD-WRKY29*) and two reporters were co-transformed into rice protoplasts as described (Zhang et al., 2011). LUC activity was quantified with a Dual-Luciferase Assay Kit (Promega, Beijing, China) according to the manufacturer’s recommendations and relative LUC activity was calculated by the ratio of LUC/Ren. Each sample was tested with three replicates.

### Yeast One-Hybrid Assays

Yeast one-hybrid analysis was performed as described in a previous report (Lin et al., 2007). We cloned the full-length coding region of *OsWRKY29* into the pB42AD vector at the *Eco*RI restriction site to produce a *pB42AD*-*OsWRKY29* construct (primer pair *pB42AD*-*OsWRKY29* in Supplementary Table S1). The promoter regions or mutant promoter regions of *OsVP1* and *OsABF1* were cloned into the pLacZi reporter vector (primer pairs listed in Supplementary Table S1) at the *Xho*I restriction site to generate *pLacZi-OsVP1*, *pLacZi*-*OsABF1*, *mpLacZi-OsVP1*, and *mpLacZi*-*OsABF1* constructs. The *pB42AD-OsWRKY29* construct was co-transformed with *pLacZi-OsVP1*, *pLacZi*-*OsABF1*, *mpLacZi-OsVP1*, and *mpLacZi*-*OsABF1* constructs into yeast strain EGY48, respectively. Transformants were cultured on SD-Trp/-Ura plates at 30°C for 3 days, followed by transfer into 5-bromo-4-chloro-3-indolyl-b-d-galactopyranoside plates for blue color development. Yeast cultures with the empty pB42AD combination with the pLacZi reporter constructs were used as negative controls.

### LUC Activity Assay

LUC activity assays were performed according to a previously described method (Duan et al., 2019). In order to generate *ProOsABF1:LUC*, *mProOsABF1:LUC*, *ProOsVP1:LUC* and *mProOsVP1:LUC* reporter constructs, we amplified ∼2 kb promoter regions of *OsABF1* and *OsVP1* using primer pairs *ProOsABF1:LUC*, *mProOsABF1:LUC*, *ProOsVP1:LUC* and *mProOsVP1:LUC* (primer pairs listed in Supplementary Table S1), and then fused them into the *Nco*I restriction site of the pGreenII 0800-LUC vector. The luciferase gene from *Renilla reniformis* (Ren) under control of the CaMV35S promoter was used as the internal control. The cDNA of *OsWRKY29* was amplified (primer pair listed in Supplementary Table S1) and inserted into the *Xba*I/*Bam*HI restriction sites of the pAN580 vector to generate a *d35S*:*OsWRKY29* effector construct. The *d35S*:*OsWRKY29* effector combined with four reporters, respectively, and the corresponding combinations were co-transformed into rice protoplasts as described (Zhang et al., 2011). Empty pAN580 vector co-transformed with the reporter constructs were used as vector controls. LUC activity was quantified with a Dual-Luciferase Assay Kit (Promega, Beijing, China) according to the manufacturer’s recommendations and relative LUC activity was calculated by the ratio of LUC/Ren. Each sample was tested in three replicates.

### ChIP *in vitro* Assays

*In vitro* ChIP assays were performed according to a published method (Li et al., 2017). Total DNA of cv. Hwayoung and purified OsWRKY29-MBP protein were used for ChIP assays. Total DNA from two-week-old seedlings was sheared into 100-500 bp fragments using an ultrasonic crusher. Full-length coding sequence of *OsWRKY29* was cloned into the expression vector pMAL-c2x (primer pairs listed in Supplementary Table S1) to generate MBP fusion protein. Expression of MBP and OsWRKY29-MBP in *Escherichia coli* strain BL21 (DE3) cells (TransGen, Beijing, China) was induced by 0.5 mM isopropylb-D-thiogalactoside at 16°C for 16 h. Fusion proteins were purified using Amylose Resin (New England BioLabs, Beijing, China) according to the manufacturer’s protocol, and protein concentrations were determined by the BSA quantitative assay. MBP and OsWRKY29-MBP were co-incubated with DNA fragments in the incubation buffer for 2 h (50 mM Tris, 1 mM EDTA, 100 mM KCl, adjusted to pH 7.0 by HCl, 5% glycerol, 0.1% Triton X-100, with freshly made 100 mM DTT added to the reaction solution to make a final concentration of 1 mM DTT). Afterward, the Amylose Resin was washed three times using the incubation buffer; 4 mL 5 M NaCl was added into for each 100 mL volume and the sample was incubated for 4 h to break down cross-linked OsWRKY29-MBP and DNA fragments. DNA fragments were subsequently collected with a DNA Recovery Kit (Zymo, Beijing, China) following the manufacturer’s recommendations. DNA fragments between 100 and 500 bp were used for ChIP-qPCR.

### ChIP-qPCR

The prepared DNA for *in vitro* ChIP assays was also used for qPCR using relevant primer pairs (Supplementary Table S1) in a SYBR Premix Ex Taq (TaKaRa) with an ABI7500 Real-Time PCR instrument. PCR were performed in triplicate for each sample, and fold enrichment was calculated against the UBQ5 gene. MBP served as a negative control.

### Electrophoretic Mobility Shift Assay

OsWRKY29 proteins (fused with maltose-binding protein [MBP] tags) were expressed in *E. coli* strain BL21 (DE3) (TransGen, Beijing, China) and affinity-purified. The probes containing the putative *OsABF1* and *OsVP1* binding sites were synthesized by Invitrogen (Shanghai) (primer pairs listed in Supplementary Table S2). The probes were labeled with biotin at the 5′ end and the competitive probes lacked the label. electrophoretic mobility shift assay (EMSA) was performed with the LightShift Chemiluminescent EMSA Kit (Thermo, No. 20148) following the manufacturer’s protocol. Briefly, equal amounts of complementary oligonucleotides were incubated at 95°C for 10 min, cooled slowly to 15°C (0.1°C/1s), and diluted to a 20 fmol final concentration. The DNA binding reaction was performed with 2 μl of probe, 2 μl Binding Buffer, 1 μg purified MBP or OsWRKY29-MBP protein, with or without competitive probes, and brought to 20 μl with added water and incubated at room temperature for 30 min. Samples were immediately applied to pre-run native polyacrylamide gel containing 6.5% acrylamide in 0.5 X Tris-Borate-EDTA buffer. After electro-blotting onto a nylon membrane (Millipore) the oligonucleotides were cross-linked using UV-light. The membrane was incubated in a blocking solution for 15 min, followed by incubation in a conjugate/blocking buffer solution for another 15 min. After intensive washing with a washing buffer, Substrate Equilibration Buffer was applied to the membrane for 5 min. Finally, Substrate Working Solution was added to visualize the signal.

## Results

### Enhanced Seed Dormancy of the *Oswrky29* Mutant

**FIGURE 1:**
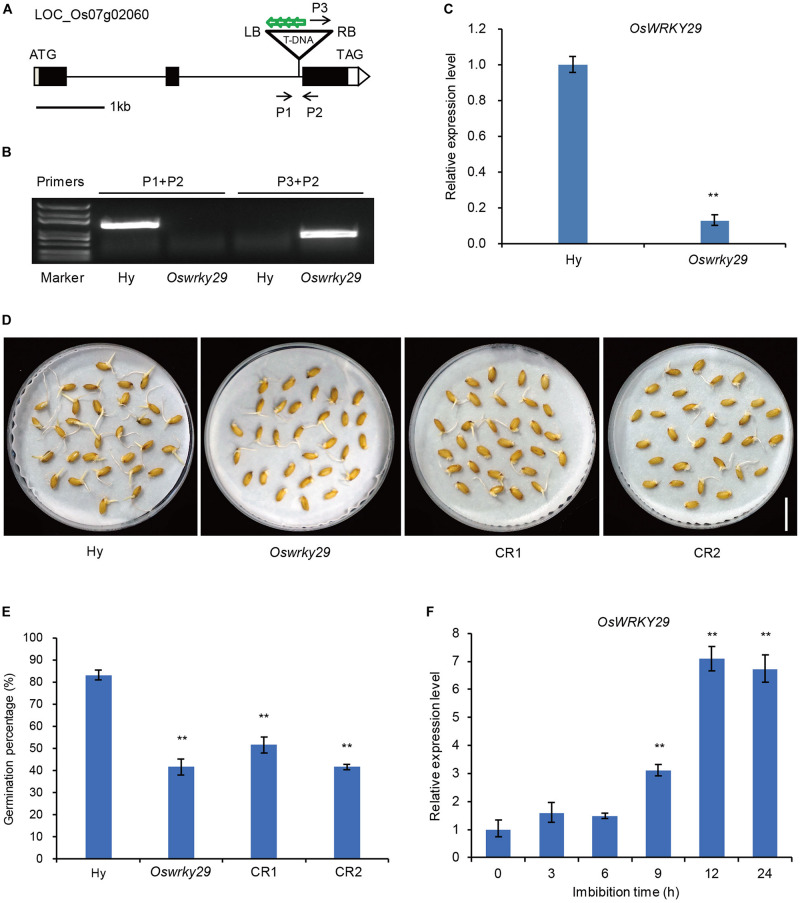
Seed dormancy phenotypes of *Oswrky29* T-DNA insertion mutant. **(A)** T-DNA insertion site in *Oswrky29* (LOC_Os07g02060) mutant is indicated by a triangle. Black boxes indicate exons, lines indicate introns, and white boxes indicate the untranslated regions. P1, P2, and P3 are the primers used to identify the T-DNA insertion site. Green arrows indicate four copies of 35S enhancers in the T-DNA insertion sequence. RB and LB indicate the right and left borders of the T-DNA insertion. **(B)** Identification of the T-DNA insertion by PCR. The primers are labeled in panel **(A)**. **(C)** RT-qPCR of the *OsWRKY29* transcript in Hwayoung (Hy) and *Oswrky29* seeds. Fresh seeds were collected at 45 days post heading for RT-qPCR analysis. Values are means ± SD (*n* = 3). The Student’s *t*-test analysis indicates a significant difference (**P < 0.01). **(D)** Seed germination phenotypes of Hy, *Oswrky29* mutant and *OsWRKY29*-*CRISPR* transgenic lines CR1 and CR2. Fresh seeds were collected at 45 days post heading for germination test. Scale bar, 2 cm. **(E)** Germination percentages for Hy, *OsWRKY29* mutant, CR1 and CR2. Fresh seeds were collected at 45 days post heading for germination test. Values are means ± SD (*n* = 3), 30 seeds were measured in each replicate. The Student’s *t*-test analysis indicates a significant difference (**P < 0.01). **(F)** Analysis of *OsWRKY29* expression levels during seed imbibition in water determined by RT-qPCR. Fresh seeds were collected at 45 days post heading and imbibition in water. RT-qPCR was performed at the indicated times. Values are means ± SD (*n* = 3). The Student’s *t*-test analysis indicates a significant difference (**P < 0.01).

A search of the T-DNA insertion database OryGenesDB^2^, showed that pGA2772 (activation-tagged T-DNA vector) was inverse-integrated in the second intron of *LOC_–_Os07g02060* in the T-DNA insertion line (Figure 1A). To confirm the T-DNA insertion site in the *Oswrky29* mutant, three primers, P1, P2, and P3 (P1 and P2 from the *OsWRKY29* genome sequence and P3 from the right border (RB) of the inserted T-DNA), were designed for PCR analysis. When P1 and P2 were used for PCR, a single band was detected only in the wild type and when P3 and P2 were used for PCR, a single band was detected in the *Oswrky29* mutant (Figure 1B). Identification of the latter sequence revealed two parts: one from the right border of the T-DNA, the other from the OsWRKY29 genome sequence (Supplementary Figure S1). These results confirmed that the mutant carried a T-DNA insertion in the second intron of *OsWRKY29*. To test if the T-DNA insertion affected the transcription of *OsWRKY29*, we performed RT-qPCR and found that the expression of *OsWRKY29* was reduced in the mutant relative to the wild type Hwayoung (Hy) (Figure 1C).

Some WRKY proteins were reported to be involved in the regulation of seed dormancy (Xie et al., 2005; Ding et al., 2014; Huang et al., 2016). To test whether OsWRKY29 also regulates seed dormancy, the fresh harvested seeds of *Oswrky29* mutant and *OsWRKY29* knockout transgenic lines (CR1 and CR2) (Supplementary Figure S2) were used to perform seed germination assays. The results showed that germination percentage of *Oswrky29* mutant was 41% compared to 83% for the wild type Hy (Figures 1D,E), suggesting that *Oswrky29* mutant had stronger seed dormancy than its wild type Hy. Consistent with this, *OsWRKY29* knockout transgenic lines (CR1 and CR2) also displayed enhanced seed dormancy (Figures 1D,E). These results showed that both the *Oswrky29* mutant and *OsWRKY29* knockout lines had stronger seed dormancy than Hy. Further, *OsWRKY29* expression was evidently decreased with the grain development (Supplementary Figure S3), but during seed imbibition, *OsWRKY29* expression was gradually upregulated (Figure 1F), indicating that the expression level of *OsWRKY29* is negatively related to seed dormancy. Taken together, our results suggest that the absence of *OsWRKY29* enhances seed dormancy.

### OsWRKY29 Functions as a Negative Regulator of Seed Dormancy

**FIGURE 2:**
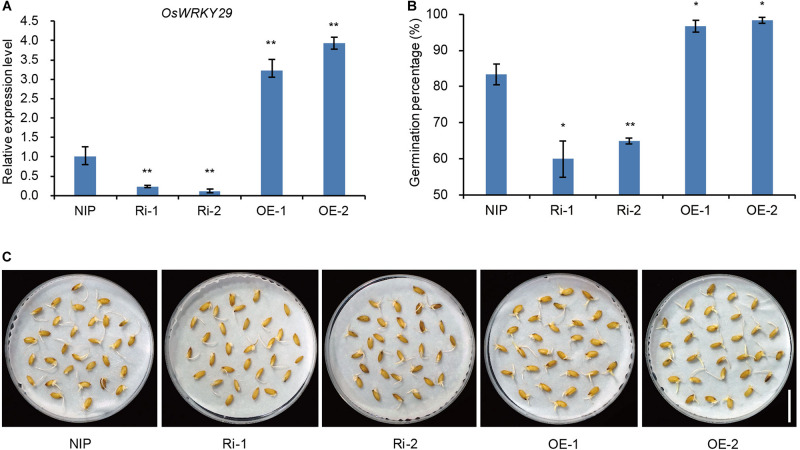
Seed dormancy phenotypes of *OsWRKY29* transgenic plants. **(A)** *OsWRKY29* expression levels in seeds of the NIP, *OsWRKY29* Ri and OE lines determined by RT-qPCR. Fresh seeds were collected at 45 days post heading for RT-qPCR analysis. Values are means ± SD (*n* = 3). The Student’s *t*-test analysis indicates a significant difference (**P < 0.01). **(B)** Seed germination percentages of NIP, *OsWRKY29* Ri and OE lines after five days of imbibition. Fresh seeds were collected at 45 days post heading for germination test. Values are means ± SD (*n* = 3), 30 seeds were measured in each replicate. The Student’s *t*-test analysis indicates a significant difference (*P < 0.05, **P < 0.01). **(C)** Seed dormancy phenotypes of NIP, *OsWRKY29* Ri and OE lines after five days of imbibition. Fresh seeds were collected at 45 days post heading for germination test. Scale bar, 2 cm.

To further confirm that knockdown of *OsWRKY29* was responsible for the *Oswrky29* mutant phenotype, two independent *OsWRKY29* RNAi lines in Nipponbare (NIP) background, Ri-1 and Ri-2, in which *OsWRKY29* transcript levels were evidently reduced, were chosen to test seed dormancy (Figure 2A). Seed germination of Ri-1 and Ri-2 was significantly reduced compared with NIP (Figures 2B,C). In contrast, the overexpression lines of *OsWRKY29*, OE-1 and OE-2, whose *OsWRKY29* transcript levels were evidently increased (Figure 2A), showed significantly higher germination percentage (almost 100%) compared to their receptor NIP (Figures 2B,C). Thus, knockdown of *OsWRKY29* apparently enhanced seed dormancy whereas its overexpression had an opposite effect, further supporting the notion that *OsWRKY29* functions as a negative regulator of seed dormancy.

### Protein Structure, Expression Pattern, and Transcription Activity of *OsWRKY29*

*OsWRKY29*, with three exons and two introns, encodes a 290 amino acid protein. By BLAST search in the NCBI database, we identified its homologs (including most reported homologs) in Arabidopsis and rice (Supplementary Figure S4A). Alignment some of these homologs using Clustal X revealed a conserved WRKYGQK motif (WRKY domain) and a C_2_H_2_ zinc-finger motif in OsWRKY29 and its homologs, suggesting that *OsWRKY29* and its homologs would bind similar DNA motifs to regulate transcription (Supplementary Figure S4B).

**FIGURE 3:**
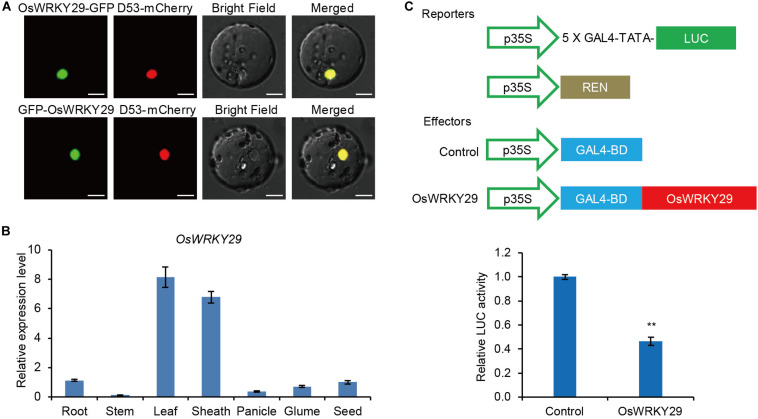
Subcellular-localization, expression pattern, and transcription activity of the *OsWRKY29* gene. **(A)** Subcellular localizations of the OsWRKY29-GFP fusion protein and GFP-OsWRKY29 fusion protein in rice protoplasts. D53-mCherry was used as the nuclear marker. Scale bar, 10 μm. **(B)** Relative expression of *OsWRKY29* in various tissues including root (2 weeks old), stems (heading stage), leaf (heading stage), leaf sheath (heading stage), panicle (2 cm), Glume (in blossom), and seed (30 DPA). Values are means ± SD (*n* = 3). **(C)** Transcription activity assay of OsWRKY29 in rice protoplasts. The reporters and effectors used in the assay were generated as shown. Firefly luciferase (LUC) and renilla luciferase (REN) activities were determined 16 h post-transformation. The relative luciferase activities in control and OsWRKY29-expressed samples were calculated by normalizing the LUC values against REN. Values are means ± SD (*n* = 3). The Student’s *t*-test analysis indicates a significant difference (**P < 0.01).

The subcellular localization analyses showed that fusion proteins OsWRKY29-GFP and GFP-OsWRKY29 were uniformly localized in the nucleus (Figure 3A), consistent with function as a transcription factor. A RT-qPCR analysis revealed that *OsWRKY29* was widely expressed in rice tissues, with the highest expression level of *OsWRKY29* transcript in leaves and lowest expression in stems (Figure 3B). To test the transcription activity of *OsWRKY29*, we performed transient dual-luciferase (LUC) assays in rice protoplasts. The result revealed that OsWRKY29 led to an obvious downregulation of relative luciferase activity compared to the control (Figure 3C). These results indicated that OsWRKY29 acts as a transcriptional repressor.

### OsWRKY29 Acts as a Repressor of ABA-Inhibited Seed Germination

Previous studies showed that WRKY transcription factors can regulate many stress responses through modulating the ABA signaling pathway. Moreover, ABA signaling was considered to be essential for regulating seed dormancy and germination (Shang et al., 2010; Tao et al., 2011). To investigate whether *OsWRKY29* regulates seed dormancy by modulating the ABA signaling pathway, we broke seed dormancy by drying seeds at 50°C for 72 h and treated seeds (dormancy released stage) of Hy, *Oswrky29* mutant and *OsWRKY29* knockout lines (CR1 and CR2) with or without ABA. Under treatment with ABA, *Oswrky29* mutant and *OsWRKY29* knockout lines showed reduced seed germination percentage relative to wild-type Hy (Supplementary Figures S5A–E), showing that *Oswrky29* mutant and *OsWRKY29* knockout lines had enhanced sensitivity to ABA. With the germination of Hy seeds, the increase in *OsWRKY29* transcript level was clearly repressed by the treatment with 3 μM ABA compared to the treatment without ABA (-ABA) (Supplementary Figure S5F). Similarly, the *OsWRKY29* transcript level was also significantly repressed by 100 μM ABA applied to Hy seedlings (Supplementary Figure S6). These observations showed that ABA can inhibit the expression of *OsWRKY29* and the lack of *OsWRKY29* enhanced sensitivity to ABA-mediated inhibition of seed germination.

**FIGURE 4:**
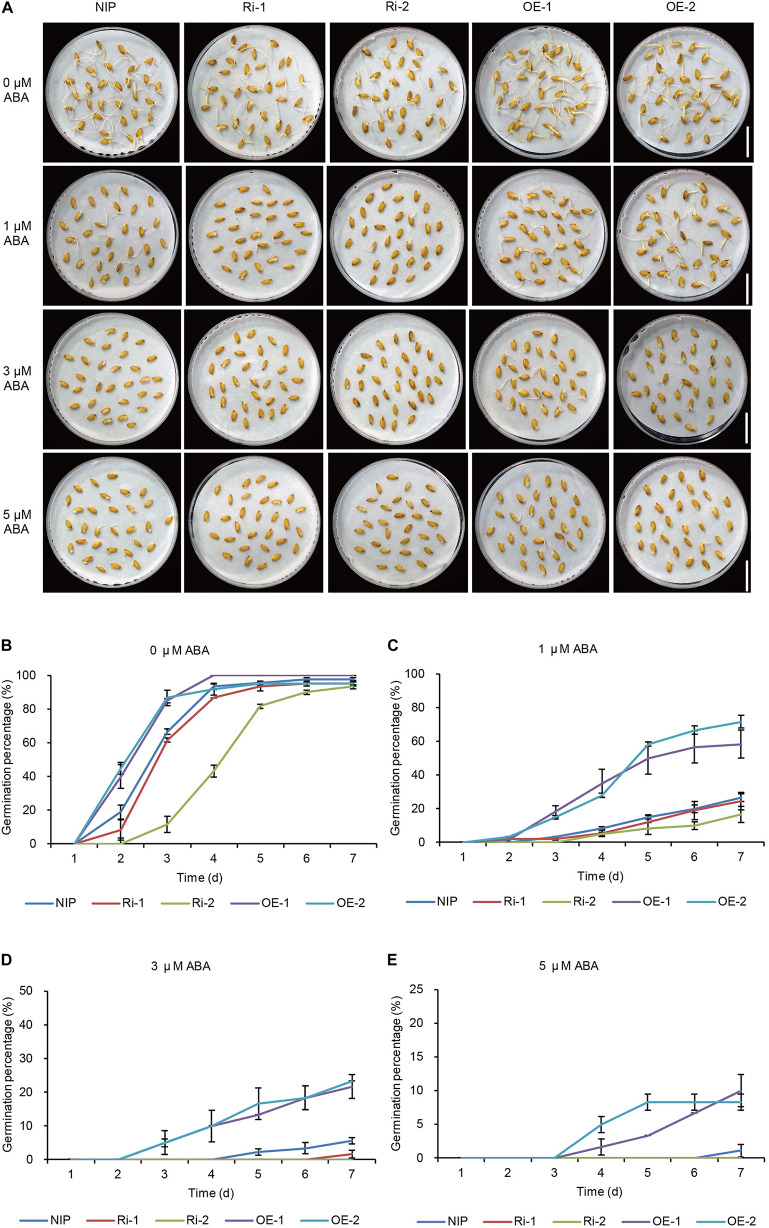
ABA responses of *OsWRKY29* RNA interference (Ri) and overexpression (OE) lines. **(A)** Seed germination phenotypes of Nipponbare (NIP), *OsWRKY29* Ri and OE lines treated with ABA at 7th day. Scale bar, 2 cm. **(B–E)** Germination time courses in water containing 0 μM ABA **(B)**, 1 μM ABA **(C)**, 3 μM ABA **(D)**, and 5 μM ABA **(E)**. Seed dormancy was broken by drying at 50°C for 72 h. The heat-treated seeds (dormancy released stage) were used for ABA treatment. Values are means ± SD (*n* = 3).

To further confirm the function of *OsWRKY29* in the ABA signaling pathway, *OsWRKY29*-overexpressing lines (OE-1, OE-2) and *OsWRKY29* RNAi lines (Ri-1, Ri-2) were also used to study the physiological role of *OsWRKY29* in ABA response. Heat-treated seeds (dormancy released stage) of *OsWRKY29*-overexpression lines and *OsWRKY29* RNAi lines were treated with or without ABA. Without the ABA treatment (0 μM ABA), seeds of *OsWRKY29*-overexpression lines displayed faster germination (Figures 4A,B) and *OsWRKY29* RNAi seeds displayed slower germination compared to NIP (Figures 4A,B). And with the ABA treatment, the germination of *OsWRKY29*-overexpression lines showed a slower decline trend in germination whereas the germination of *OsWRKY29* RNAi lines showed a faster decline trend relative to NIP (Figures 4C–E). Knockdown of *OsWRKY29* enhances while the overexpression of *OsWRKY29* reduces ABA sensitivity during seed germination especially by 3 μM ABA treatment (Supplementary Figure S7). These data indicated that OsWRKY29 is a negative regulator in ABA-inhibited seed germination.

### OsWRKY29 Negatively Regulates Several ABA-Related Genes

**FIGURE 5:**
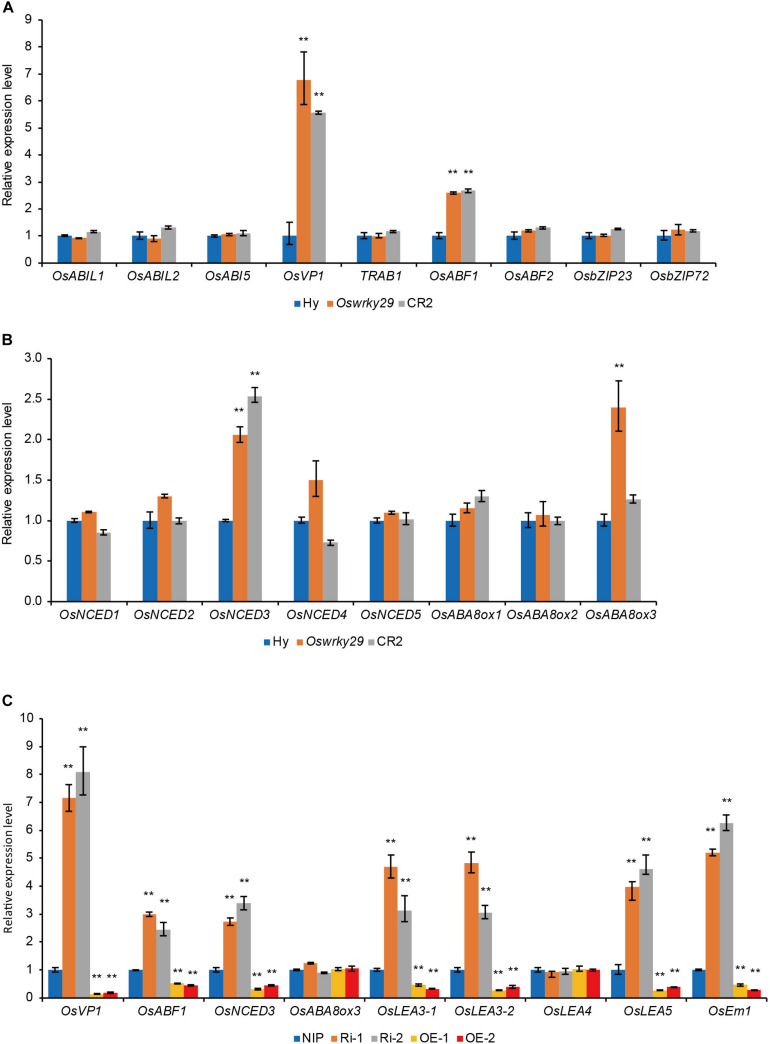
The expression levels of ABA-related genes. **(A)** Relative transcript levels of ABA response genes in seeds of Hy, *Oswrky29* and CR2. Fresh seeds were collected at 45 days post heading for RT-qPCR analysis. Values are means ± SD (*n* = 3). The Student’s *t*-test analysis indicates a significant difference (**P < 0.01). **(B)** Relative transcript levels of ABA synthesis and metabolism genes in seeds of Hy, *Oswrky29* and CR2. Fresh seeds were collected at 45 days post heading for RT-qPCR analysis. Values are means ± SD (*n* = 3). The Student’s *t*-test analysis indicates a significant difference (**P < 0.01). **(C)** Relative transcript levels of several ABA-related genes in seeds of NIP, *OsWRKY29* Ri lines and *OsWRKY29* OE lines. Fresh seeds were collected at 45 days post heading for RT-qPCR analysis. Values are means ± SD (*n* = 3). The Student’s *t*-test analysis indicates a significant difference (**P < 0.01).

To understand the role of OsWRKY29 in ABA-mediated seed dormancy, expression levels of several ABA-related genes were analyzed in seeds of WT, *Oswrky29* mutant and CR2, including ABA response genes, *OsABI-LIKE1* (*OsABIL1*) (Li et al., 2015), *OsABI-LIKE2* (*OsABIL2*) (Li et al., 2015), *OsABI5* (Zou et al., 2008), *OsVP1* (Hobo et al., 1999; Miyoshi et al., 2002), *TRAB1* (Hobo et al., 1999; Kobayashi et al., 2005), *OsABF1* (Hossain et al., 2010), *OsABF2* (Yang et al., 2011; Tang et al., 2012), *OsbZIP23* (Xiang et al., 2008), and *OsbZIP72* (Lu et al., 2009), and ABA synthesis and metabolism genes, *OsNCED1*, *OsNCED2*, *OsNCED3*, *OsNCED4*, *OsNCED5*, *OsABA8ox1*, *OsABA8ox2*, and *OsABA8ox3* (Zhu et al., 2009). Expression levels of positive ABA responsive factors, *OsVP1* and *OsABF1*, and ABA synthesis gene *OsNCED3*, were higher in both *Oswrky29* and CR2, and ABA metabolism gene *OsABA8ox3*, was only higher in *Oswrky29* (Figures 5A,B). However, expression levels of other ABA-related genes, including *OsABIL1*, *OsABIL2*, *OsABI5*, *TRAB1*, *OsABF1*, *OsABF2*, *OsbZIP23*, *OsbZIP72*, *OsNCED1*, *OsNCED2*, *OsNCED4*, *OsNCED5*, *OsABA8ox1*, and *OsABA8ox2* showed no evident difference among WT, *Oswrky29* and CR2 (Figures 5A,B). We further verified that the transcript levels of *OsVP1*, *OsABF1* and *OsNCED3* were upregulated in *OsWRKY29* Ri lines but downregulated in *OsWRKY29* OE lines compared with their receptor NIP (Figure 5C). Interestingly, the expression level of *OsABA8ox3* showed no difference among NIP, Ri and OE lines. In addition, the expression levels of some late embryogenesis abundant protein (*LEA*) genes (*OsLEA3-1*, *OsLEA3-2*, *OsLEA5* and *OsEm1*) were higher in Ri lines and lower in OE lines, compared to NIP (Figure 5C). These results suggest that *OsWRKY29* negatively regulates several ABA-related genes.

### OsWRKY29 Suppresses Directly *OsABF1* and *OsVP1* Expression

**FIGURE 6:**
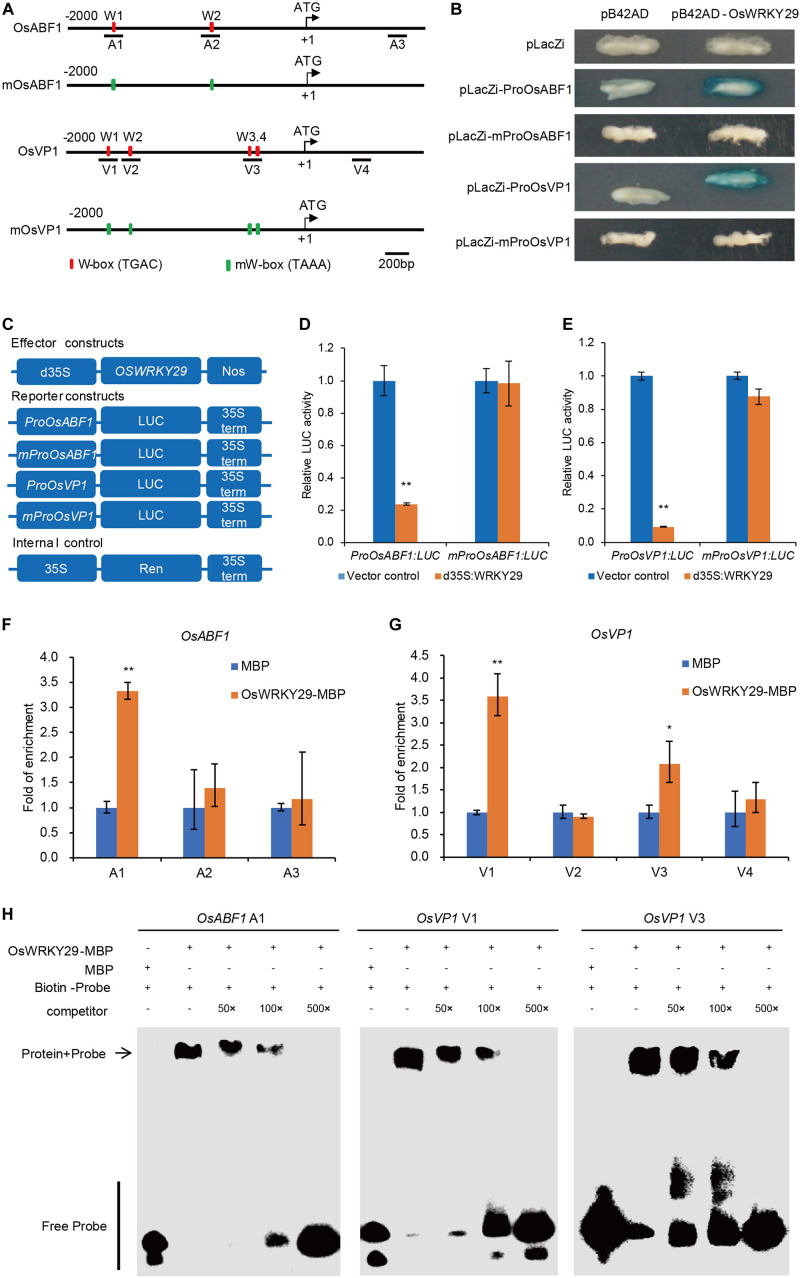
OsWRKY29 represses *OsABF1* and *OsVP1* expression by binding to their promoters. **(A)** Schematic indicating sequences 2 kb upstream of the start sites and parts of the coding sequences of *OsABF1* and *OsVP1*. The translational start sites (ATG) shown at position + 1. Red rectangles represent W-boxes. Green rectangles represent mW-boxes (TGAC mutated to TAAA). The numbers (A1–A3 and V1–V4) indicate the tested regions. **(B)** Yeast one-hybrid assays showing OsWRKY29 binding to the promoters of *OsABF1* and *OsVP1*. **(C)** Schematic diagram of the effector and reporter constructs. Full-length coding region of OsWRKY29 under control of a double 35S promoter was used as the effector. The firefly luciferase gene LUC driven by the *OsABF1* and *OsVP1* promoters and mutant promoters. The Renilla luciferase gene *Ren* driven by the 35S promoter were used as the reporter and internal control, respectively. **(D,E)** Relative LUC activities of *ProOsABF1:LUC* and *mProOsABF1:LUC* reporters **(D)** and *ProOsVP1:LUC* and *mProOsVP1:LUC* reporters **(E)** after co-expression with *35S:OsWRKY29* in rice protoplasts. 35S empty vector was used as control. Relative LUC activity was calculated by LUC/Ren. Values are means ± SD (*n* = 3). The Student’s *t*-test analysis indicates a significant difference (**P < 0.01). **(F,G)** ChIP-qPCR assays shows the target DNA fragments bound by OsWRKY29-MBP. OsWRKY29-MBP and MBP alone were incubated with total rice DNA for 4 h, pulled down, washed, and subjected to qPCR for *OsABF1* **(F)** and *OsVP1* **(G)**. Fold enrichment was normalized against the ubiquitin promoter. Values are means ± SD (*n* = 3). The Student’s *t*-test analysis indicates a significant difference (*P < 0.05, **P < 0.01). **(H)** EMSA analysis showing the binding of recombinant OsWRKY29 protein to the promoters of *OsABF1* (A1) and *OsVP1* (V1 and V3).

Previous studies demonstrated that WRKY proteins regulate expression of their target genes by binding to W-boxes in their promoters. Analyses of 2 kb promoter sequences identified two W-boxes in the *OsABF1* promoter, four W-boxes in *OsVP1* promoter and one W-box in the *OsNCED3* promoter (Figure 6A and Supplementary Figures S8, S9A). We hypothesized that OsWRKY29 binds to the W-boxes of the promoters of *OsABF1*, *OsVP1*, and *OsNCED3* to regulate their expression.

To test the above inference, we first performed yeast one-hybrid (Y1H) assays to evaluate if OsWRKY29 directly binds to the promoters of *OsABF1*, *OsVP1*, and *OsNCED3*. As shown in Figure 6B and Supplementary Figure S9, the color of the control was unchanged whereas all experimental groups except *OsNCED3* turned blue, suggesting that OsWRKY29 directly binds to the promoters of *OsABF1* and *OsVP1* but not *OsNCED3*. Then, we mutated these W-boxes from TGAC to TAAA in this experiment, and found that OsWRKY29 can’t bind to the mutated promoters of *OsABF1* and *OsVP1* (Figure 6B), suggesting W-boxes are necessary for OsWRKY29 to bind the promoter of *OsABF1* or *OsVP1*. We further performed transient dual-luciferase (LUC) assays in rice protoplasts to test the transcriptional regulatory relationship between OsWRKY29 and *OsABF1* or *OsVP1*. As shown in Figures 6C–E, OsWRKY29 greatly repressed the expression of the luciferase (LUC) reporter gene driven by the *OsABF1* or *OsVP1* promoter; but when the W-boxes were changed from TGAC to TAAA, the inhibition activity of OsWRKY29 on the mutated promoter of *OsABF1* and *OsVP1* was disappeared, indicating that OsWRKY29 inhibits the transcription activity of the *OsABF1* and *OsVP1* dependent on W-boxes.

We also performed an *in vitro* qPCR assay after chromatin immunoprecipitation (ChIP-qPCR). OsWRKY29 enriched on A1 promoter region of *OsABF1* as well as the V1 and V3 promoter regions of *OsVP1* rather than other promoter regions of *OsABF1* and *OsVP1* (Figures 6F,G), suggesting that OsWRKY29 could specifically bind to the A1 region of *OsABF1* promoter or V1 and V3 regions of *OsVP1* promoter. Furthermore, the EMSA showed that OsWRKY29-MBP recombinant proteins bound to the DNA probes in the A1 region of *OsABF1* promoter or both the V1 and V3 regions of *OsVP1* promoter. Moreover, unlabeled competing probes could effectively reduce the binding ability of OsWRKY29 in a dose-dependent manner (Figure 6H). Taken together, these results support the view that OsWRKY29 represses *OsABF1* and *OsVP1* expression by binding to the W-box region in the promoter of *OsABF1* or *OsVP1* in rice.

## Discussion

### *OsWRKY29* Negatively Regulate ABA Signaling During Seed Dormancy

In this study, we identified a dormancy enhancement T-DNA insertion mutant *Oswrky29* in rice (Figures 1A–E). The T-DNA insertion within the second intron of *OsWRKY29* can reduced its expression level, which was responsible for the enhanced dormancy of *Oswrky29* mutant (Figure 1). In addition, *OsWRKY29* expression is negatively related to seed dormancy during seed development or seed germination (Figure 1F and Supplementary Figures S3, S5F) and its expression was inhibited by ABA in germinating seeds and early seedlings (Supplementary Figures S5F, S6). As a member of the WRKY transcription factor family, OsWRKY29, including a WRKY domain and a C_2_H_2_ type zinc-finger motif (Supplementary Figure S4B), is localized in the nucleus and acts as a transcriptional repressor (Figures 3A,C). ABA sensitivity assays indicated that *OsWRKY29* likely represses seed dormancy through regulation of ABA signaling pathway (Figure 4 and Supplementary Figures S5, S7). Further assays showed that OsWRKY29 inhibits seed dormancy likely by repressing the expression of ABA positive response genes such as *OsABF1* and *OsVP1* by binding to their promoters (Figures 5, 6).

**FIGURE 7:**
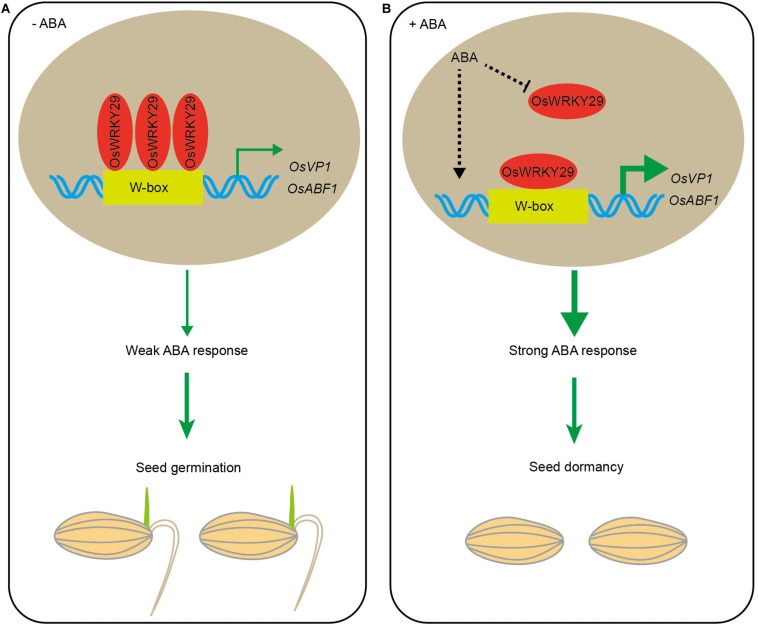
Hypothetical model for regulation of seed dormancy by *OsWRKY29*. **(A)** In the absence of ABA, OsWRKY29 binds to the *OsVP1* and *OsABF1* promoters to repress their expression, thus weakening the ABA response and permitting seed germination. **(B)** The presence of ABA represses *OsWRKY29* expression to relieve its repression on the expression of *OsVP1* and *OsABF1*, thus resulting in the high expression of *OsVP1* and *OsABF1* to strengthen ABA response and promote seed dormancy.

In summary, these results suggest that, in the absence of ABA, OsWRKY29 binds to the promoters of *OsVP1* and *OsABF1* (positive ABA response factors) to repress their expression, thus weakening ABA-mediated repression of seed germination. The presence of ABA represses *OsWRKY29* expression, then releasing its repression on the expression of *OsVP1* and *OsABF1*, thus permitting higher expression of *OsVP1* and *OsABF1* to enhance seed dormancy (Figure 7).

### *OsWRKY29* Probably Regulates Stress Response and Plant Development

WRKY transcription factors are reported to be involved in regulation of stress response through ABA signaling. In Arabidopsis, *abo3*, a T−DNA insertion mutant of *AtWRKY63*, is hypersensitive to ABA, impairs ABA-induced stomatal closure, and has lower drought tolerance during both seedling establishment and growth (Ren et al., 2010). In rice, *OsWRKY45* alleles play different roles in regulating ABA signaling and salt stress but have similar roles in response to drought and cold: *OsWRKY45-1* negatively and *OsWRKY45-2* positively regulate ABA signaling; however, *OsWRKY45-2* but not *OsWRKY45-1* negatively regulates the response of rice to salt stress (Tao et al., 2011). It was shown here that *OsWRKY29* is also involved in ABA signaling by directly regulating expression of *OsVP1* and *OsABF1*, suggesting that *OsWRKY29* is probably involved in ABA-mediated regulation of stress response. Previous studies indicated that *OsABF1* is a universally positive regulator of drought tolerance; *abf1* mutants are more sensitive to salinity and drought treatments (Zhang et al., 2017). *OsABF1* also regulates transition to floral development in rice in response to drought stress by directly activating gene *OsWRKY104* (Zhang et al., 2016). As an upstream regulator of *OsABF1*, *OsWRKY29* might also be involved in regulation of drought tolerance and flowering.

### OsWRKY29 Might Have a Balancing Effect on ABA Signaling

Some WRKY transcription factors function as promoters of ABA signaling by transcriptional activation and inhibition activities. For example, *Atwrky2* knockout mutants reduced the expression of some ABA-responsive genes *ABI3, ABI5, Em1*, and *Em6*; conversely, ABA-induced *AtWRKY2* accumulation requires *ABI5*, *ABI3*, *ABA2* and *ABA3* during seed germination, indicating a positive feedback loop between *AtWRKY2* and *ABI3* or *ABI5* in regulating ABA response (Jiang and Yu, 2009). Induction of *AtWRKY63* transcription by ABA was impaired in *abi1*, *abi2*, and *abi5* lines and the *abo3* mutation reduced the expression of *ABF2*, *RD29A and COR47* (Ren et al., 2010). *AtWRKY41* was reported to promote ABA signaling via direct regulation of *ABI3* transcript levels (Ding et al., 2014). The transcript of *AtWRKY6* was repressed during seed germination but induced by exogenous ABA; and vice versa, AtWRKY6 promoted ABA signaling by directly downregulating expression of *RAV1* (Huang et al., 2016). OsWRKY72 and OsWRKY77 promote ABA-induced *HVA22* expression in barley aleurone cells (Xie et al., 2005). Some WRKY proteins also act as repressors of ABA signaling. For example, AtWRKY18, AtWRKY40 and AtWRKY60 regulate ABA response by a de-repression mechanism by which the Mg-chelatase H subunit / putative ABA receptor (ABAR) recruits the WRKY proteins (which function as negative regulators of ABA signaling) from the nucleus to chloroplast and releases downstream ABA response genes, such as *MYB2*, *ABI4*, *ABF4*, *ABI5*, *DREB1A*, and *RAB18* (Shang et al., 2010). OsWRKY24 and OsWRKY45 repress the induction of ABA to *HVA22* in barley aleurone cells (Xie et al., 2005).

Differing from the positive feedback loop between *AtWRKY2* and ABA signaling, *OsWRKY29* was inhibited by ABA in germinating seeds and early seedlings (Supplementary Figures S5F, S6), suggesting a negative feedback loop between OsWRKY29 and the ABA signaling pathway. To verify if the molecular mechanism of OsWRKY29 was similar to those of AtWRKY18, AtWRKY40 and AtWRKY60, we investigated the subcellular localization of OsWRKY29 when treated with ABA and found that it was still localized in the nucleus (Supplementary Figure S10). Therefore, the repressive mechanism of OsWRKY29 on ABA signaling is probably different from that of AtWRKY18, AtWRKY40, and AtWRKY60.

Previous studies showed that WRKY proteins are key nodes in ABA-responsive networks: some WRKY proteins are negative regulators and others act as positive regulators of ABA response by promoting or inhibiting downstream ABA response genes. Other studies have shown that excessively strong or weak ABA responses can disrupt the balance between plant growth and stress response (Ding and De Smet, 2013; Skubacz et al., 2016; Yang et al., 2019). Taken together, these findings suggest that plants can recruit ABA response promoters such as OsWRKY72 and OsWRKY77 or repressors such as OsWRKY29 from an array of WRKY transcription factors to balance ABA signal responses for optimal growth and development.

## Accession Numbers

Sequence data from this article can be found in the EMBL/GenBank data libraries under the following accession numbers: OsWRKY29, Os07g0111400; OsABIL1, Os01g0583100; OsABIL2, Os05g0592800; OsABI5, Os01g0859300; OsVP1, Os01g0911700; TRAB1, Os08g0472000; OsABF1, Os01g0867300; OsABF2, Os06g0211200; OsbZIP23, Os02g0766700; OsbZIP72, Os09g0456200; OsNCED1, Os02g0704000; OsNCED2, Os12g04- 35200; OsNCED3, Os03g0645900; OsNCED4, Os07g0154100; OsNCED5, Os12g0617400; OsABA8ox1, Os02g0703600; OsAB- A8ox2, Os08g0472800; OsABA8ox3, Os09g0457100; OsLEA- 3-1, Os05g0542500; OsLEA3-2, Os03g0322900; OsLEA4, Os06g0110200; OsLEA5, Os05g0584200; OsEm1, Os05g0584200; OsWRKY16, Os01g0665500; OsWRKY49, Os05g0565900; OsW- RKY11, Os01g0626400; OsWRKY8, Os05g0583000; AtWRKY28, At4g18170; AtWRKY8, At5g46350; AtWRKY48, At5g49520; AtWRKY43, At2g46130; AtWRKY13, At4g39410; OsWRKY34, Os02g0652100; OsWRKY7, Os05g0537100; OsWRKY77, Os01- g0584900; AtWRKY2, At5g56270; OsWRKY24, Os01g0826400; AtWRKY63, At1g66600; OsWRKY45, Os05g0322900; AtWRK- Y41, At4g11070; AtWRKY6, At1g62300; AtWRKY40, At1g8- 0840; AtWRKY18, At4g31800; AtWRKY60, At2g25000; OsW- RKY72, Os11g0490900; AtWRKY45, At3g01970; UBQ5, and Os03g0234200.

## Data Availability Statement

All datasets generated for this study are included in the article/Supplementary Material.

## Author Contributions

JW supervised the project. CZ, LJ, and JW designed the research. CZ, JL, TZ, XL RM, CM, and TN performed the experiments. JW, XiaoZ, LZ, XingZ, and QW provided the technical assistance. CZ, QL, and LJ analyzed the data and wrote the manuscript. XinZ and XG generated the transgenic plants. SL cultivated the transgenic plants in the field. QL, LJ, and JW revised the manuscript. All authors have read and approved the final manuscript.

## Conflict of Interest

The authors declare that the research was conducted in the absence of any commercial or financial relationships that could be construed as a potential conflict of interest.

## Footnotes

1 www.genscript.com/tools/real-time-pcr-taqman-primer-design-tool

2 http://orygenesdb.cirad.fr/
