# Supplementary material for: WRKY Transcription Factor OsWRKY29 Represses Seed Dormancy in Rice by Weakening Abscisic Acid Response
Source: Front Plant Sci. 2020 May 27;11:691. doi: 10.3389/fpls.2020.00691 (PMC7268104; doi:10.3389/fpls.2020.00691)
Supplement: Supplementary file 1 [file Data_Sheet_1.doc]

**Supplementary Material**

**Supplementary Figure S1**


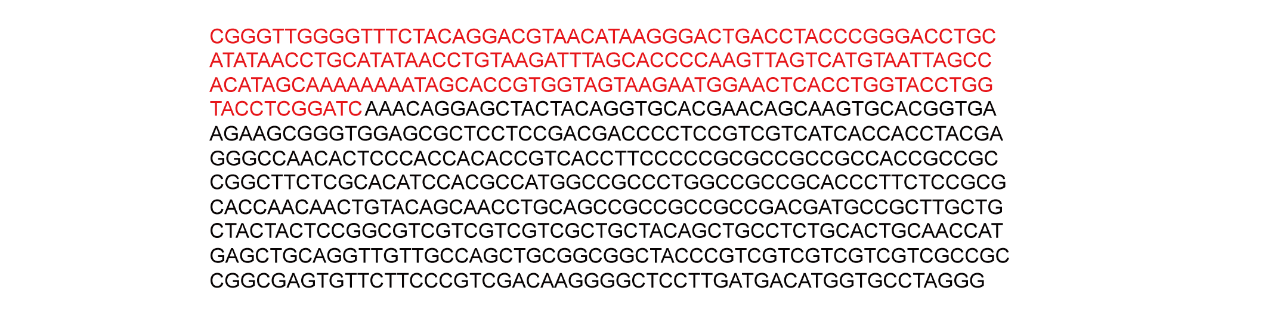


Supplementary Figure S1. Sequence amplified by primers P3 and P2. The sequence in red font is from the right border of the T-DNA insertion vector; the sequence in black font is from the *OsWRKY29* gene.

**Supplementary Figure S2**


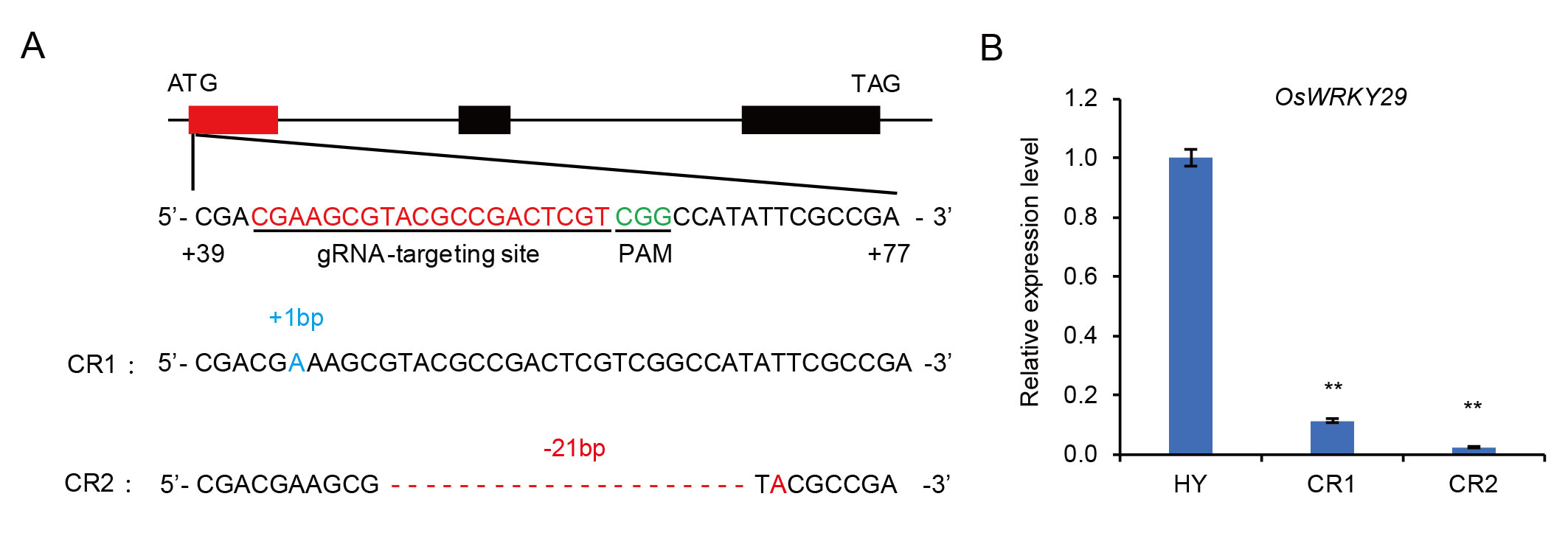


Supplementary Figure S2. Identification of *OsWRKY29* knockout mutants. (A) Schematic of the CRISPR/Cas9-mediated edit in the first exon of *OsWRKY29*. The gRNA-targeted site and PAM are indicated in red and green font, respectively. Red dashed line represents deleted nucleotides. Added and replaced bases are highlighted in blue and red fonts, respectively. (B) RT-qPCR of the *OsWRKY29* transcript in Hwayoung (Hy) and *OsWRKY29* knockout mutant (CR1 and CR2) seeds. Seeds were collected at 45 days post heading for RT-qPCR analysis. Values are means ± SD (n=3). The Student’s *t*-test analysis indicates a significant difference (***P* <0.01).

**Supplementary Figure S3**


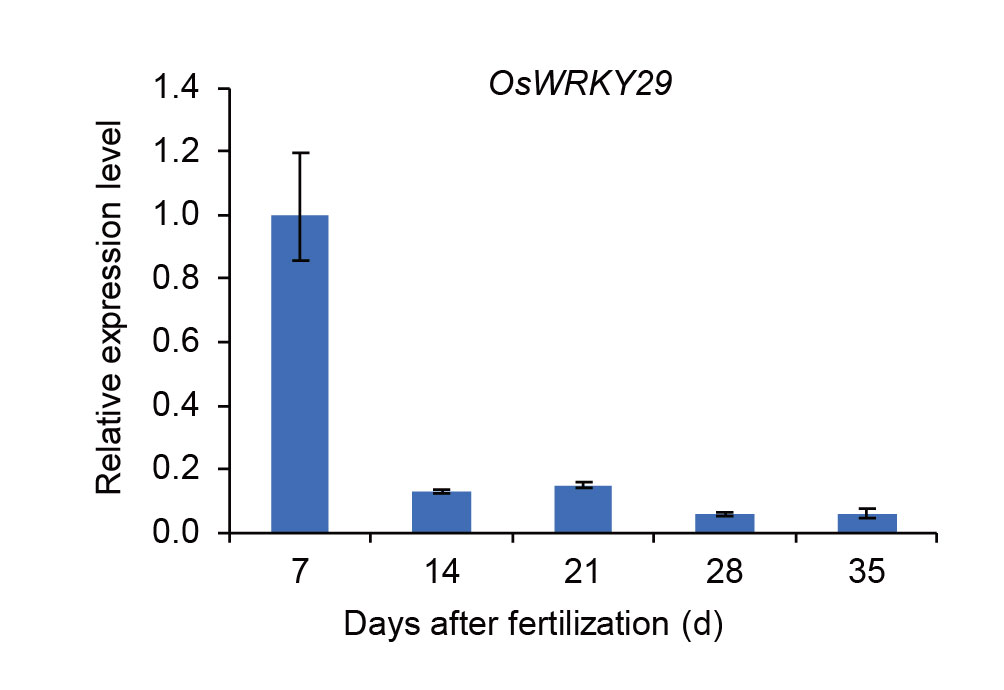


Supplementary Figure S3. Analysis of *OsWRKY29* expression levels during seed development by RT-qPCR. Seeds were collected at the indicated times for RT-qPCR analysis. Values are means ± SD (n=3)

**Supplementary Figure S4**


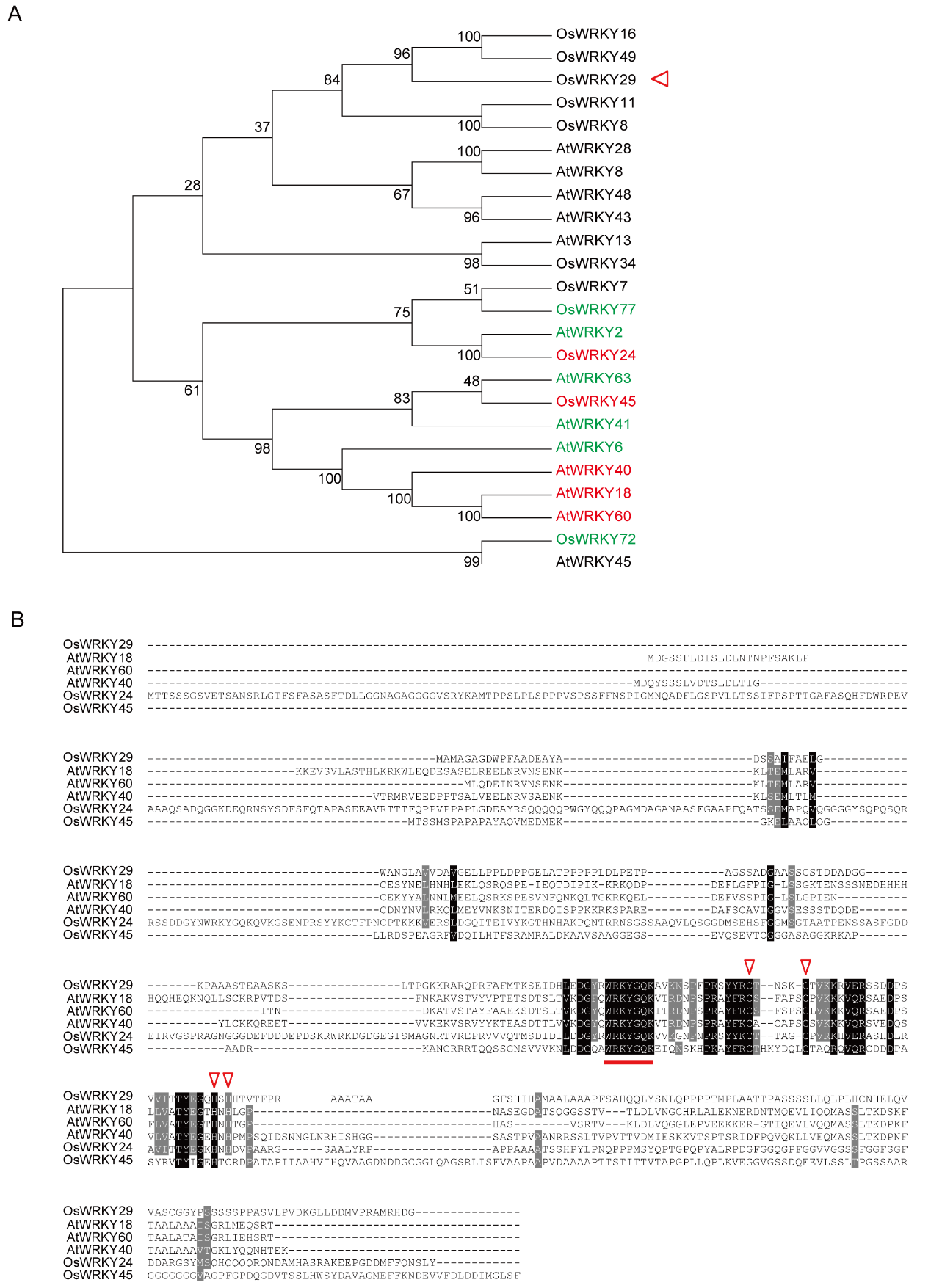


Supplementary Figure S4. Homology analysis of OsWRKY29. (A) Phylogenic tree of OsWRKY29 and its homologues. OsWRKY29 is indicated by a red triangle. The promoters and inhibitors of ABA signaling are highlighted in green and red fonts, respectively. (B) Amino acid sequence similarity between OsWRKY29 and its homologues. Identical and similar residues are displayed in black and gray, respectively. The WRKYGQK motif is underlined and the C_2_H_2_ zinc finger motif is marked with red triangles.

**Supplementary Figure S5**

**
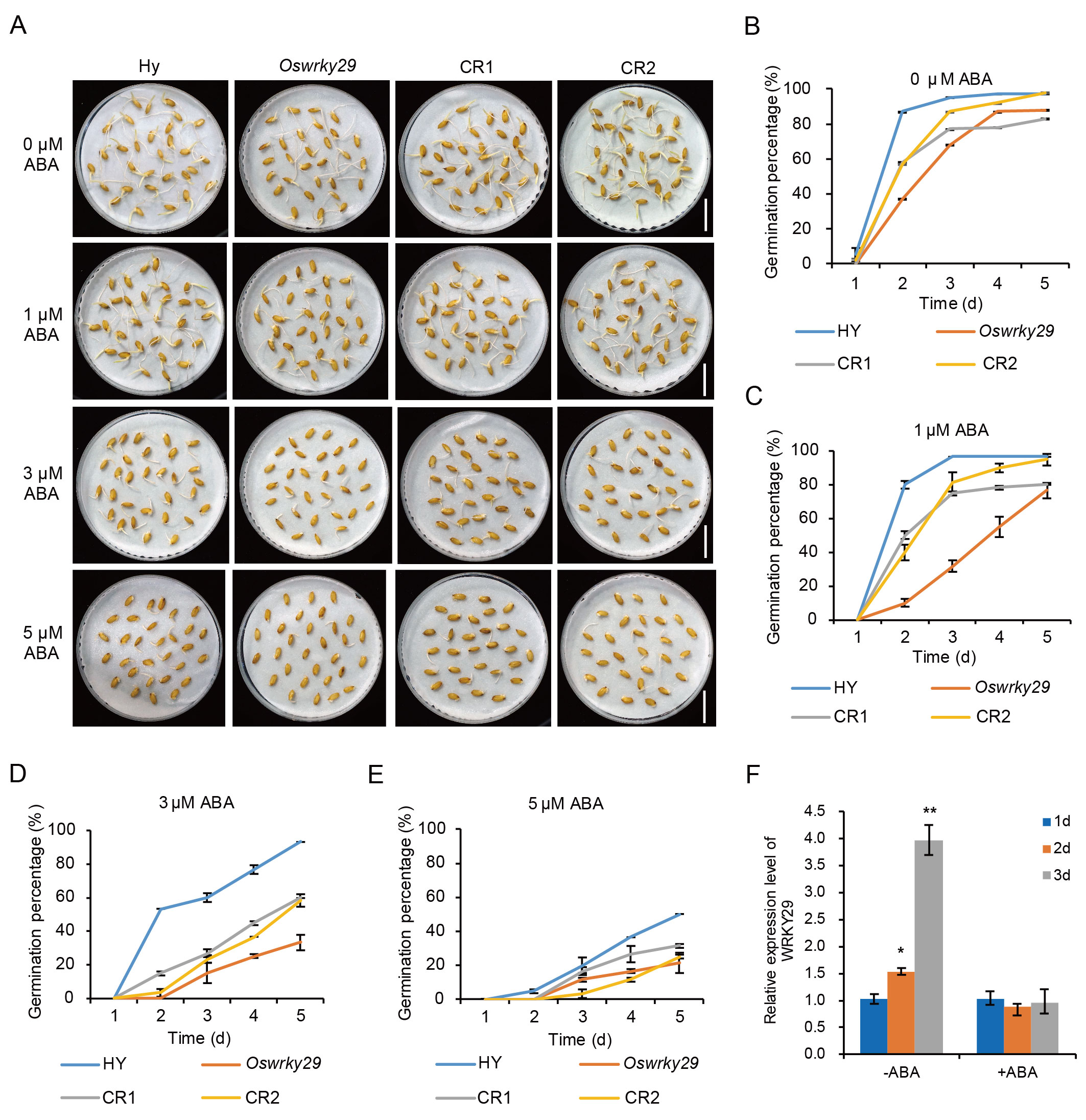
**

Supplementary Figure S5**.** ABA sensitivity of *Oswrky29* mutant and *OsWRKY29*-*CRISPR* lines. (A) Seed germination phenotypes of Hwayoung (Hy), *Oswrky29* mutant and *OsWRKY29*-*CRISPR* lines (CR1 and CR2) treated with ABA for 5 days. Scale bar, 2 cm. (B-E) Germination time courses in water containing 0 µM ABA (B), 1 µM ABA (C), 3 µM ABA (D) and 5 µM ABA (E). Seeds were dried at 50 °C for 72 hours to break seed dormancy and then used for ABA treatment. Values are means ± SD (n=3). (F) Expression of *OsWRKY29* in germinating seeds of Hy treated with or without 3 µM ABA. RT-qPCR was performed at the indicated times. Values are means ± SD (n=3). The Student’s *t*-test analysis indicates a significant difference (**P* <0.05, ***P* <0.01).

**Supplementary Figure S6**


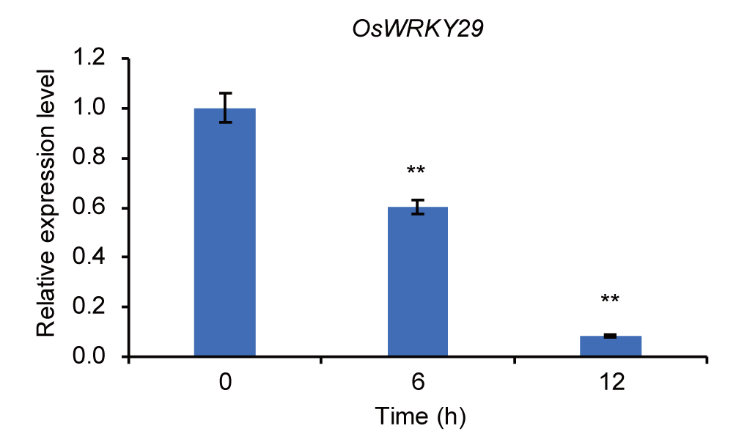


Supplementary Figure S6. ABA represses expression of *OsWRKY29* in cv. Hwayoung seedlings. Seven-day-old seedlings were treated with 100 µM ABA for 6 h and 12 h, and then sampled for RT-qPCR analysis. Values are means ± SD (n=3). The Student’s *t*-test analysis indicates a significant difference (***P* <0.01).

**Supplementary Figure S7**


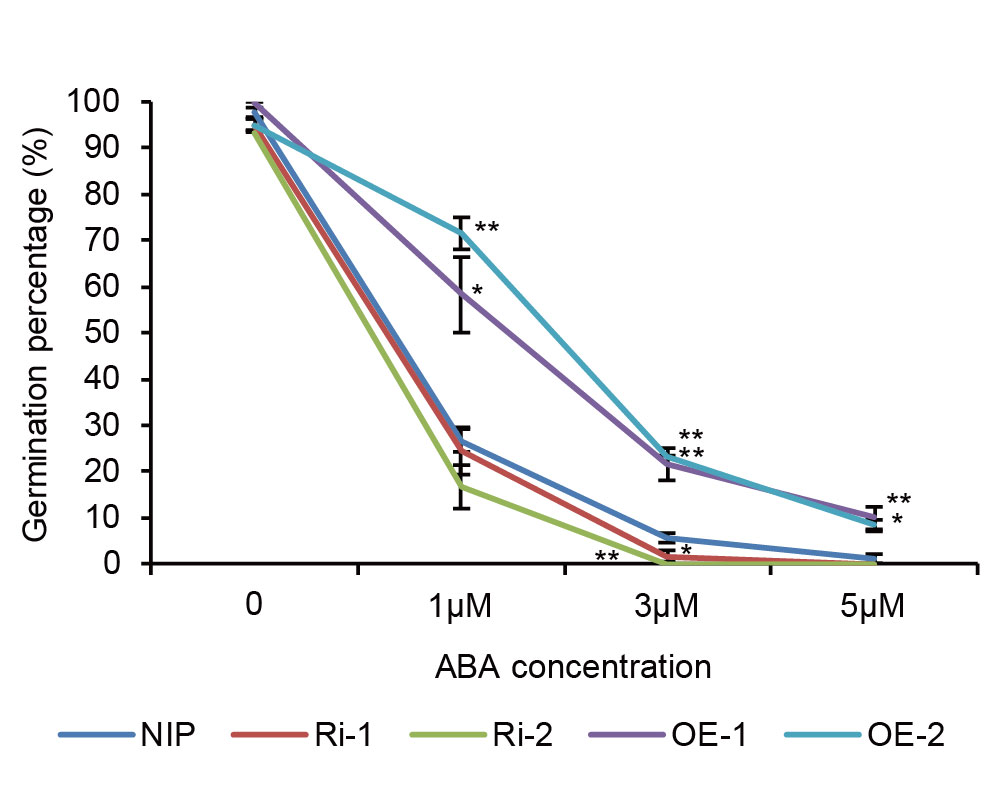


Supplementary Figure S7. Knockdown of *OsWRKY29* enhances while the overexpression of *OsWRKY29* reduces ABA sensitivity during seed germination. Seeds were dried at 50 °C for 72 hours to break seed dormancy and then used for ABA treatment. Germination data was collected on the 7th day. Values are means ± SD (n=3). The Student’s *t*-test analysis indicates a significant difference (**P* <0.05, ***P* <0.01).

**Supplementary Figure S8**


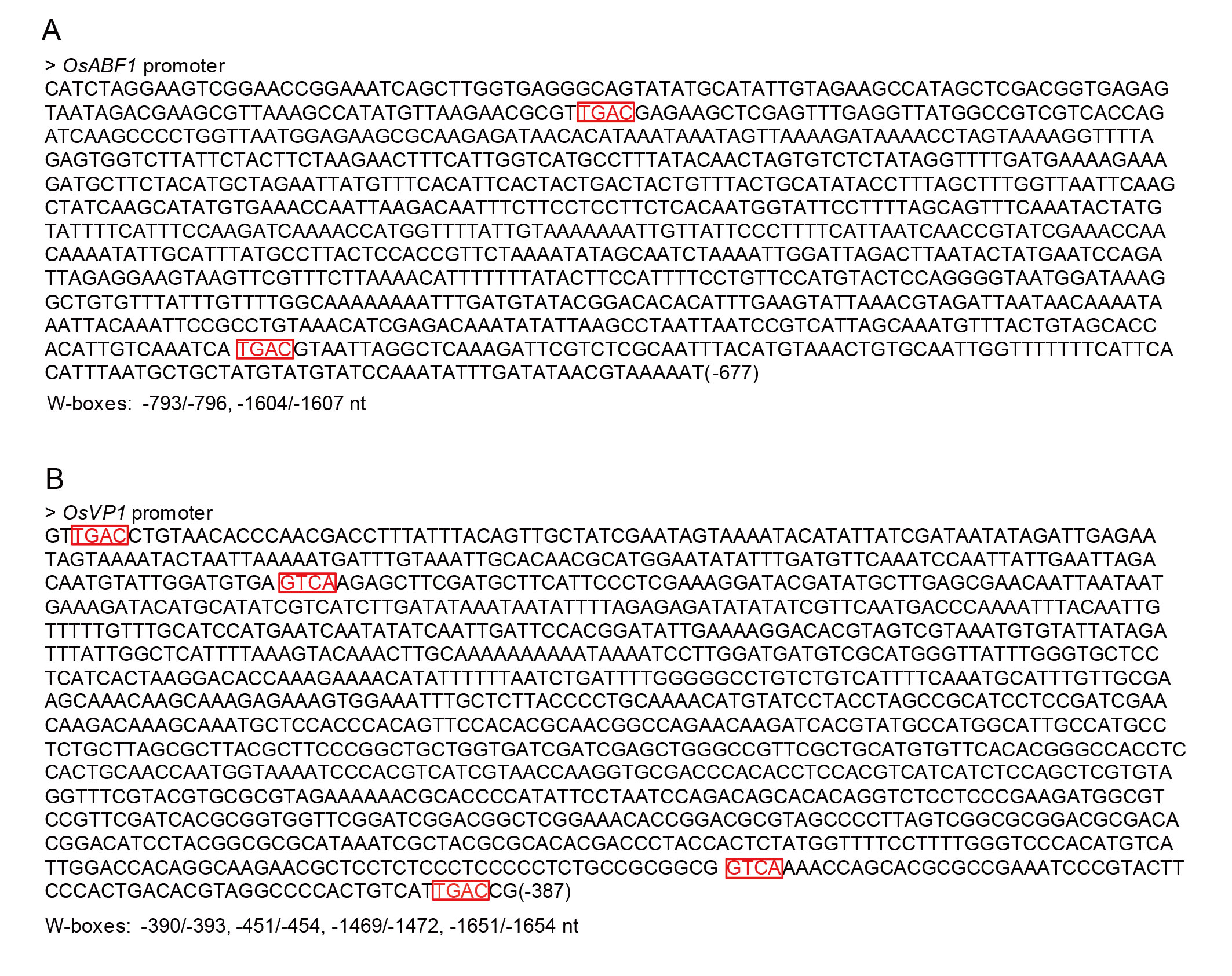


Supplementary Figure S8. W-boxes within the promoter regions of *OsABF1* and *OsVP1*. The OsWRKY29 binding motifs (W-boxes) are boxed. The positions of binding sites are shown below. nt, nucleotide.

**Supplementary Figure S9**


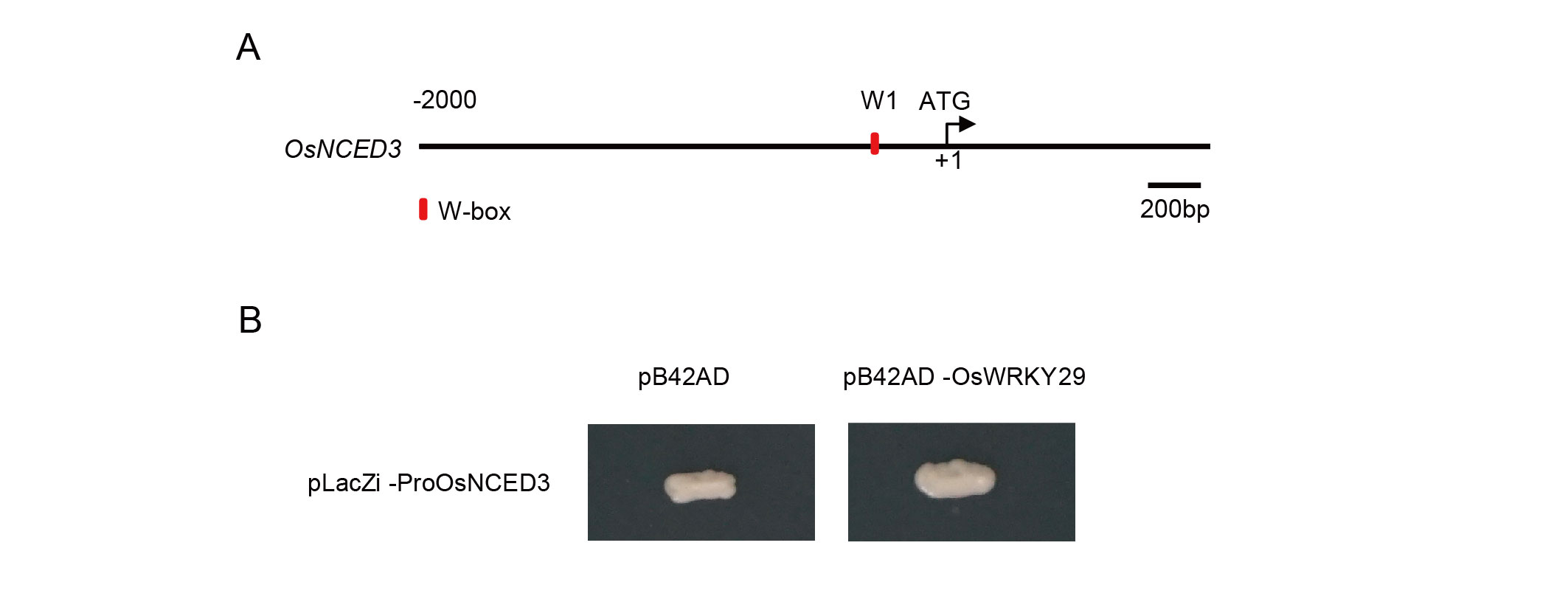


Supplementary Figure S9. OsWRKY29 does not bind to the promoter of *OsNCED3*. (A) The sequence including 2 kb upstream of the start site and parts of the coding sequence of *OsNCED3* are shown in the schematic. The translational start site (ATG) is shown at position +1. Red rectangle represents the position of the W-box. (B) Yeast one-hybrid assay showing that OsWRKY29 does not bind to the promoter of *OsNCED3*. About 2 kb promoter of *OsNCED3* was fused to the upstream region of the pLacZi reporter gene for testing the binding ability of OsWRKY29 to the W-box. Empty pB42AD (left) was used as the negative control.

**Supplementary Figure S10**


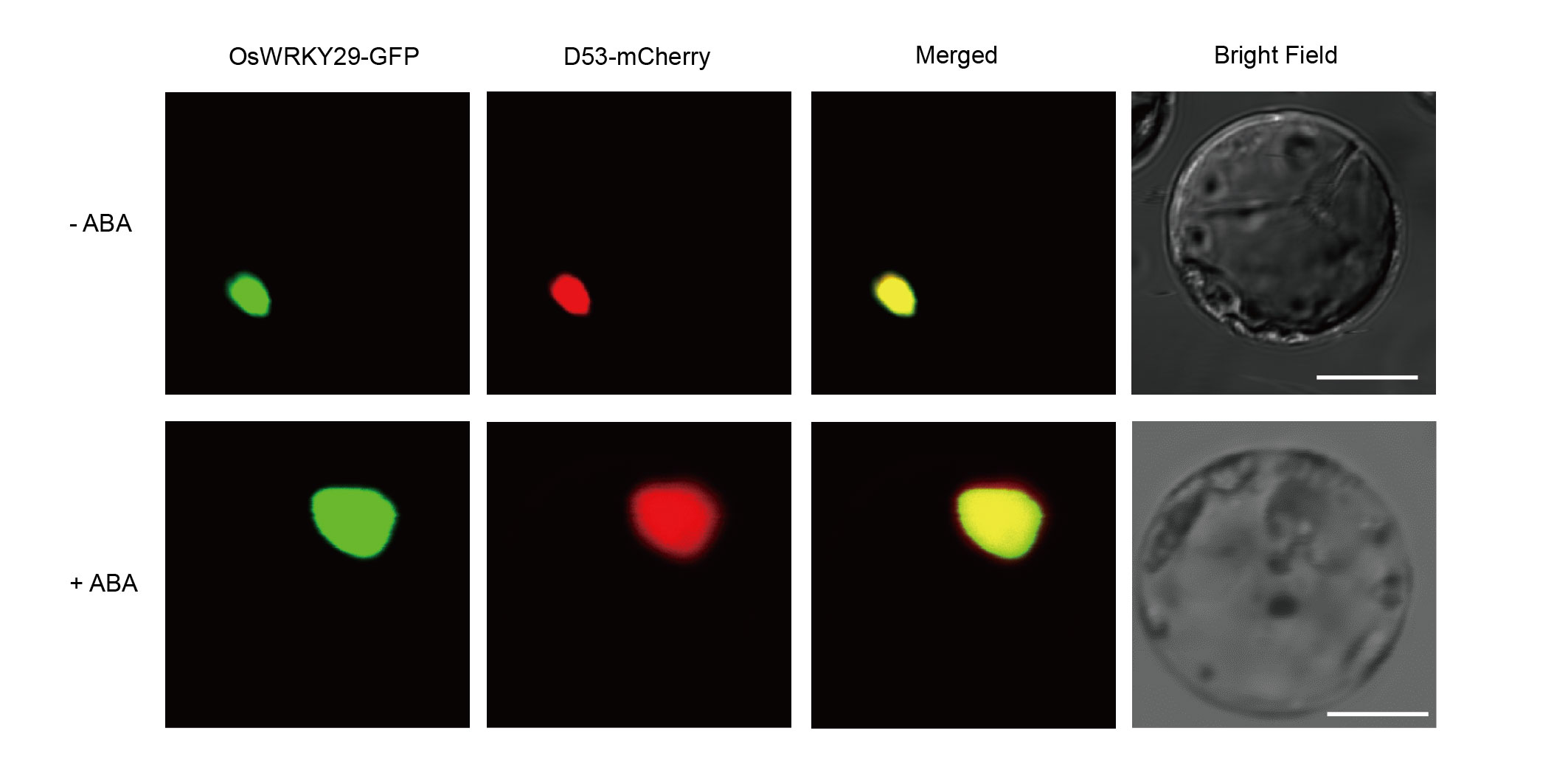


Supplementary Figure S10. Subcellular localization of the OsWRKY29-GFP fusion protein in rice protoplast with or without 3 µM ABA treatment. D53-mCherry was used as the nuclear marker. Bar, 5 µm.

**Supplementary Table 1.** List of the primers used in this study

| Primer name | Sequence (5ʹ-3ʹ) |
| --- | --- |
| P1 | ATTTCGGTGAGCCACATACG |
| P2 | ACTACCCATCATGCCTCATT |
| P3 | CCACAGTTTTCGCGATCCAGACTG |
| UBQ5-F | AACCAGCTGAGGCCCAAGA |
| UBQ5-R | ACGATTGATTTAACCAGTCCATGA |
| RT-OsWRKY29-F | CGGCTTCTCGCACATCCA |
| RT-OsWRKY29-R | AGCAGCAAGCGGCATCG |
| RT-OsABIL1-F | TGCCGCTATCAGTGGATCAT |
| RT-OsABIL1-R | CGACATGGCAAGAACACCAA |
| RT-OsABIL2-F | AAGACGAGTACGCGAGGATT |
| RT-OsABIL2-R | GACAACTGTGACCTCAGGGA |
| RTOsABI5-F | GCAGGAGGAAGCGGTTTATG |
| RTOsABI5-R | AGTGTTACCTGTCGGTCGTT |
| RT-OsVP1-F | ACATAGACCAGCTCCTCGAC |
| RT-OsVP1-R | GGACATGGCGTCCTCTATCA |
| RT-TRAB1-F | CCGGCTGTCAACAACAGCTC |
| RT-TRAB1-R | GAAACCCATCGCACCAGCAG |
| RT-OsABF1-F | ACCTGATGGATCCGATGGA |
| RT-OsABF1-R | ATCTTGGCGTTCTCCTCCTC |
| RT-OsABF2-F | GGCGGATGATCAAGAACAGG |
| RT-OsABF2-R | TGGTCCAAGTTGCTGAGTGA |
| RT-OsbZIP23-F | GAGATCACGCTGGAGGAGTT |
| RT-OsbZIP23-R | ACCATCGGAGGGAACACATT |
| RT-OsbZIP72-F | TGTATTCGCTGACGTTCGAC |
| RT-OsbZIP72-R | GAGCAGCTCGTCCATGTTC |
| RTOsNCED1-F | CTACATCCCTCCTGCTGCTT |
| RTOsNCED1-R | CTACCAACTGCTCGTCCTCT |
| RTOsNCED2-F | TTCCAAGGTACGCCAAGGAT |
| RTOsNCED2-R | TCTCCTCTCCATCAGCCTCT |
| RTOsNCED3-F | TTCGCCATCACCGAGAACTA |
| RTOsNCED3-R | GAGCATCTCCTGGAGCTTGA |
| RTOsNCED4-F | GTCCAAGCCGTACCTCAAGT |
| RTOsNCED4-R | TCCTGGAGCTTGAACACGAT |
| RTOsNCED5-F | AGGTGTGGCAAGAAGAAGGA |
| RTOsNCED5-R | GCACATTCGTGATGAACCCT |
| RTOsABA8ox1-F | GTCATCGGCGTCATCTTCGC |
| RTOsABA8ox1-R | TGACCCGGCTCGTCATCTTC |
| RTOsABA8ox2-F | CTGCTCACCGACGACCAGAT |
| RTOsABA8ox2-R | CCCTCGTTGGCCACGTAGAT |
| RTOsABA8ox3-F | TGGCCCTAACCCACAAGGTG |
| RTOsABA8ox3-R | CGGCATCACCTTCCATCCCT |
| RTOsLEA3-1-F | ATACCAAGGAGGCGACGAAG |
| RTOsLEA3-1-R | GTGCTGGAGGTCTTGTCCTT |
| RTOsLEA3-2-F | AAGAACAAGCTGGGCGAGTA |
| RTOsLEA3-2-R | CTTGAACTCCGTCGCCTTC |
| RTOsLEA4-F | CCGACGAGAAGAAGGAGGTG |
| RTOsLEA4-R | TTATGGAGCCTGTGCTGGAT |
| RTOsLEA5-F | GCTCACCTACACCCTCAAGT |
| RTOsLEA5-R | GATCTTGGCCGGTATCTCCA |
| RTOsEm1-F | AGACGAGGAAGGAGCAGAT |
| RTOsEm1-R | GGACTTGGTCTTGTACTTG |
| OsWRKY29-CRISPR-F | AGATGATCCGTGGCACGAAGCGTACGCCGACTCGTGTTTTAGAGCTATGC |
| OsWRKY29-CRISPR-R | GCATAGCTCTAAAACACGAGTCGGCGTACGCTTCGTGCCACGGATCATCT |
| OsWRKY29-RNAi-SacI-F | TTCTGCACTAGGTACCAGGCCTGGGATGGCTATCGCTGGAGA |
| OsWRKY29-RNAi-SacI-R | CTGACGTAGGGGCGATAGAGCTCGCGTGGATGTGCGAGAA |
| OsWRKY29-RNAi-SnaBI-F | CGGGGATCCGTCGACTACGGATGGCTATCGCTGGAGA |
| OsWRKY29-RNAi-SnaBI-R | AGGTGGAAGACGCGTTACGCGTGGATGTGCGAGAA |
| OsWRKY29-OX-F | TTACTTCTGCACTAGGTACCATGGCCATGGCGGGCGCCGGC |
| OsWRKY29-OX-R | GAATTCCCGGGGATCCCTACCCATCATGCCTCATTG |
| OsWRKY29-GFP-F | CGGAGCTAGCTCTAGAATGGCCATGGCGGGCGCCGGCG |
| OsWRKY29-GFP-R | TGCTCACCATGGATCCCCCATCATGCCTCATTGCC |
| GFP-OsWRKY29-F | CGAGCTGTACAGATCTATGGCCATGGCGGGCGCCGGCG |
| GFP-OsWRKY29-R | CGAGCTGTACAGATCTCTACCCATCATGCCTCATTGCC |
| GAL4-BD-OsWRKY29-F | GGGGACAAGTTTGTACAAAAAAGCAGGCTTCATGGCCATGGCGGGCGCCG |
| GAL4-BD-OsWRKY29-R | GGGGACCACTTTGTACAAGAAAGCTGGGTCCTACCCATCATGCCTCATT |
| pB42AD-WRKY29-F | TGCCTCTCCCGAATTCATGGCCATGGCGGGCGCCGGC |
| pB42AD-OsWRKY29-R | CGAGTCGGCCGAATTCCTACCCATCATGCCTCATTG |
| pLacZi-OsVP1-F | ATCTGTCGACCTCGAGATTTTCCAGATTTATTCGTG |
| pLacZi-OsVP1-R | GAGCACATGCCTCGAGTTACTGAGAAAGGAGACGA |
| Placzi-mOsVP1-F | ATCTGTCGACCTCGAGTaAaCTGTAACACCCAACGA |
| Placzi-mOsVP1-R | GAGCACATGCCTCGAGTGTAACGTTTAATGACAGTG |
| pLacZi-OsABF1-F | ATCTGTCGACCTCGAGAGGATAGGGAAATAGAGGAA |
| pLacZi-OsABF1-R | GAGCACATGCCTCGAGAGAGCGTGATTAAGTGGAT |
| Placzi-mOsABF1-F | ATCTGTCGACCTCGAGAACGCGTTaAaGAGAAGCTC |
| Placzi-mOsABF1-R | GAGCACATGCCTCGAGTAATTACTTTATGATTTGAC |
| pLacZi-OsNCED3-F | ATCTGTCGACCTCGAGGCGAACTGACGTGGAGGA |
| pLacZi-OsNCED3-R | GAGCACATGCCTCGAGGACGAACGCTGAGGCTTG |
| ProOsABF1-LUC-F | AGATCGAATTCCATGGAGGATAGGGAAATAGAGGAA |
| ProOsABF1-LUC-R | TTGGCGTCTTCCATGGAGAGCGTGATTAAGTGGAT |
| mProOsABF1-LUC-F | AGATCGAATTCCATGGAACGCGTTaAaGAGAAGCTC |
| mProOsABF1-LUC-R | TTGGCGTCTTCCATGGTAATTACTTTATGATTTGAC |
| ProOsVP1-LUC-F | AGATCGAATTCCATGGATTTTCCAGATTTATTCGTG |
| ProOsVP1-LUC-R | TTGGCGTCTTCCATGGTTACTGAGAAAGGAGACGA |
| mProOsVP1-LUC-F | AGATCGAATTCCATGGTaAaCTGTAACACCCAACGA |
| mProOsVP1-LUC-R | TTGGCGTCTTCCATGGTGTAACGTTTAATGACAGTG |
| OsWRKY29-MBP-F | TTCAGAATTCGGATCCATGGCCATGGCGGGCGCCG |
| OsWRKY29-MBP-R | CGACTCTAGAGGATCCTTACCCATCATGCCTCATTGCC |
| CHIP-OsABF1-1F | ATAGCTCGACGGTGAGAGTA |
| CHIP-OsABF1-1R | CCATAACCTCAAACTCGAGC |
| CHIP-OsABF1-2F | CTGTAAACATCGAGACAAAT |
| CHIP-OsABF1-2R | CATAGCAGCATTAAATGTGA |
| ChIP-OsABF1-3F | TAGGTGAGTAGGCATGTGAC |
| ChIP-OsABF1-3R | GCATCCTAAGCAGATGGCTA |
| CHIP-OsVP1-1F | GTTATGCCAAAACAACATAG |
| CHIP-OsVP1-1R | TACTATTCGATAGCAACTGT |
| CHIP-OsVP1-2F | CGCATGGAATATATTTGATG |
| CHIP-OsVP1-2R | TCAAGCATATCGTATCCTTT |
| CHIP-OsVP1-3F | ATTGGACCACAGGCAAGAAC |
| CHIP-OsVP1-3R | GTAACGGTCAATGACAGTGG |
| CHIP-OsVP1-4F | AAGAGCAGCGTGGTCGTGAG |
| CHIP-OsVP1-4R | AATTGCTGCGGCCAGGCCAT |

**Supplementary Table 2.** Oligonucleotides used for EMSA assays

| Probe name | Sequence (5ʹ-3ʹ) |
| --- | --- |
| Biotin-EMSA-OsABF1-A1 | AGCGTTAAAGCCATATGTTAAGAACGCGTTGACGAGAAGCTCGAGTTTGAGGTTATGGCC |
| Biotin-EMSA-OsVP1-V1 | TCAACCTACCATATCGCTCAAATGATTGTTGACCTGTAACACCCAACGACCTTTATTTAC |
| Biotin-EMSA-OsVP1-V3 | CGGTCAAAACCAGCACGCGCCGAAATCCCGTACTTCCCACTGACACGTAGGCCCCACTGTCATTGACCG |
| EMSA-OsABF1-A1 | AGCGTTAAAGCCATATGTTAAGAACGCGTTGACGAGAAGCTCGAGTTTGAGGTTATGGCC |
| EMSA-OsVP1-V3 | CGGTCAAAACCAGCACGCGCCGAAATCCCGTACTTCCCACTGACACGTAGGCCCCACTGTCATTGACCG |
| EMSA-OsVP1-V1 | TCAACCTACCATATCGCTCAAATGATTGTTGACCTGTAACACCCAACGACCTTTATTTAC |
